# Effect of different treatment modalities on ovarian cancer patients with liver metastases: A retrospective cohort study based on SEER

**DOI:** 10.1371/journal.pone.0299504

**Published:** 2024-04-18

**Authors:** Na Li, Shanxiu Jin, Jingran Wu, Hongjuan Ji, Cheng Du, Bona Liu

**Affiliations:** 1 Department of Gynecology and Obstetrics, First Affiliated Hospital, Jilin University, Jilin, P. R. China; 2 Department of Oncology, General Hospital of Northern Theater Command, Dalian Medical University, Shenyang, P. R. China; 3 Department of Oncology, General Hospital of Northern Theater Command, Jinzhou Medical University, Shenyang, P. R. China; 4 Department of Oncology, General Hospital of Northern Theater Command, Shenyang, P. R. China; University of Brescia: Universita degli Studi di Brescia, ITALY

## Abstract

**Background:**

To examine the trends in morbidity and mortality among ovarian cancer patients with liver metastases, and investigate the impact of different treatments on both overall survival (OS) and cancer-specific survival (CSS).

**Methods:**

2,925 ovarian cancer patients with liver metastases from Surveillance, Epidemiology, and End Results 2010–2019 were included. The primary endpoint was considered as OS and CSS. We conducted trend analysis of the incidence, OS and CSS rates of liver metastases in ovarian cancer. Univariate and multivariate COX proportional risk models were used to investigate the association between different treatment methods and OS, and univariate and multivariate competing risk models were employed to evaluate the impact of treatment methods on CSS.

**Results:**

At the end of follow-up, 689 patients remained alive. The OS and CSS rates were 76.44% and 72.99% for all patients, respectively. There was a significant decreasing trend in the incidence [average annual percent change (AAPC) = -2.3, 95% confidence interval (CI): -3.9, -0.7], all-cause mortality (AAPC = -12.8, 95% CI: -15.6, -9.9) and specific mortality (AAPC = -13.0, 95% CI: -16.1, -9.8) rate of liver metastases in ovarian cancer. After adjusting all confounding factor, only receiving surgery was associated with OS [hazard ratio (HR) = 0.39, 95%CI: 0.31–0.48]/CSS (HR = 0.37, 95%CI: 0.30–0.47). Chemotherapy was found to be protective factor for OS (HR = 0.33, 95%CI: 0.30–0.37)/CSS (HR = 0.44, 95%CI: 0.39–0.50) of ovarian cancer patients, while not receiving surgery remained a risk factor. Additionally, the result of subgroup analyses also showed that only receiving surgery and chemotherapy still were significant protective factor of OS and CSS for patients without other distant metastases, with distant metastases to the bone, lung, brain or other organs, with bone metastasis, and with lung metastasis.

**Conclusion:**

Our research has elucidated a downward trend in morbidity and mortality rates among patients with liver metastases originating from ovarian cancer. Only receiving surgery and chemotherapy as therapies methods confer survival benefits to patients.

## Introduction

Ovarian cancer is a prevalent gynecological malignancy and a leading cause of mortality in females [1]. Ovarian cancer possesses the potential to spread through tissue, lymph system, and blood [2]. Approximately 70% of ovarian cancer patients present with distant metastases at the time of diagnosis, resulting in an overall 5-year survival rate of less than 30% [3]. Epidemiological investigation indicates that the liver is the most frequent site of distant metastasis in ovarian cancer, followed by distant lymph nodes, lung, bone and brain [4]. The median survival time among patients with liver metastases was only 30 months [5], indicating a poor prognosis for ovarian cancer patients with liver metastases.

Previous studies have indicated that patients with advanced ovarian cancer who undergo debulking surgery have a more favorable prognosis compared to those who do not receive this treatment [6, 7]. The amount of residual disease following debulking surgery is a significant prognostic indicator for patients [8]. One study based on Surveillance, Epidemiology, and End Results (SEER) database showed that radiotherapy may be associated with a poorer prognosis in patients with primary ovarian cancer compared to those who do not receive radiation therapy [9]. In the study of Teckie et al., radiotherapy was considered an efficacious treatment for brain metastases of epithelial ovarian cancer [10]. In addition, prolonged delay in the initiation of adjuvant chemotherapy was associated with a decrease in overall survival (OS) rates for patients with advanced ovarian cancer [11]. However, to the best of our knowledge, the prognostic implications of various therapies on ovarian cancer patients with liver metastases remain unknown. It is imperative to investigate the optimal treatment for ovarian cancer patients with liver metastasis in order to enhance patient outcomes and alleviate disease burden.

Herein, the aim of this study was to examine the trends in morbidity and mortality among ovarian cancer patients with liver metastases using data from the SEER database, and to investigate the impact of different treatments on both OS and cancer-specific survival (CSS) in this population.

## Methods

### Population selection

This retrospective cohort study used data from the SEER database. The SEER database is a free access database, which compiles comprehensive information on cancer patients in the United States, encompassing demographics, tumor characteristics and details regarding mortality and survival rates [12, 13]. In this study, SEER*Stat software (version 8.4.0) was utilized to identify patients diagnosed with primary ovarian cancer from the SEER database 2010–2019.

**Fig 1:**
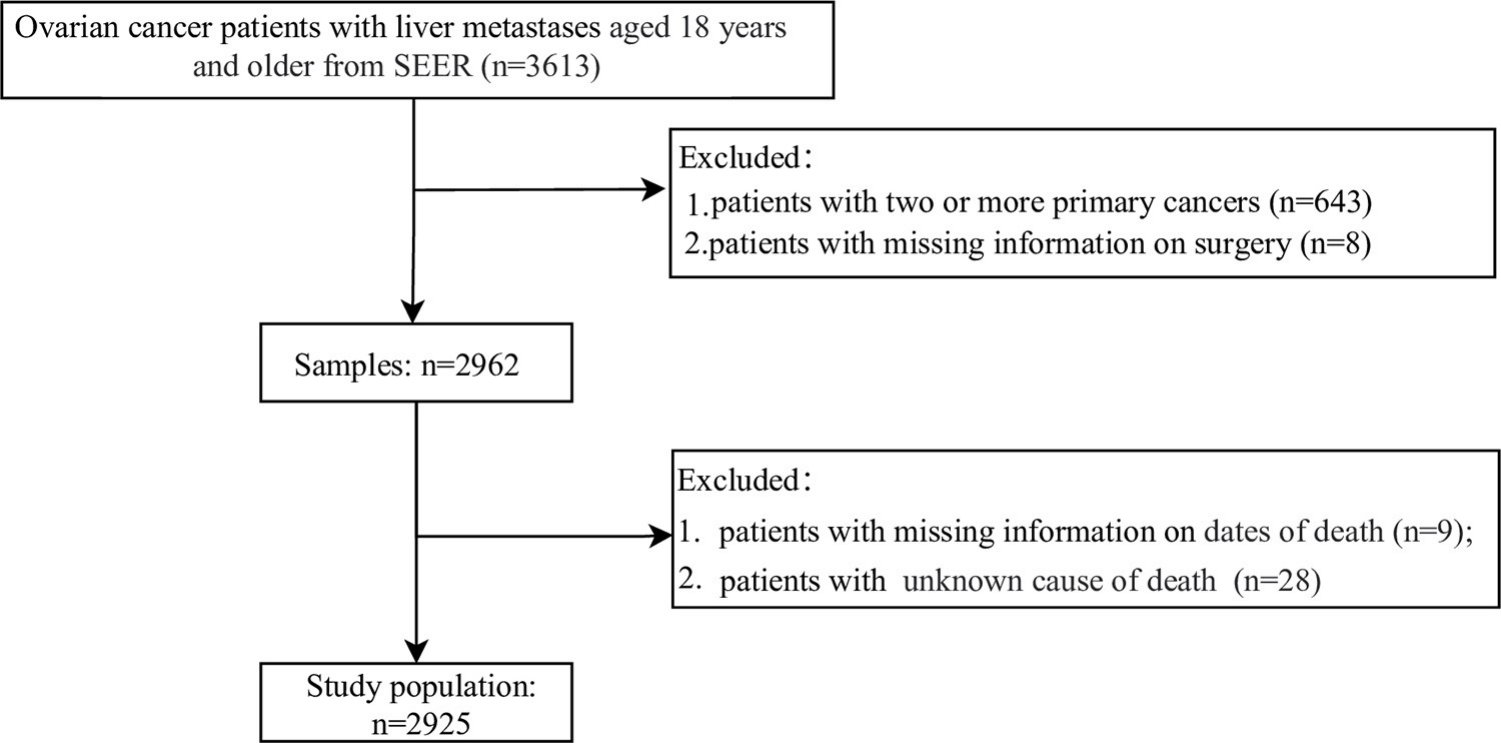
The process of selecting participants.

The study population was required to meet the following inclusion criteria: (1) diagnosis of primary ovarian cancer based on International Classification of Diseases for Oncology codes (ICD-O-3); (2) age at diagnosis of 18 years or older; and (3) presence of liver metastases at the time of diagnosis. The exclusion criteria included: (1) patients with two or more primary cancers; (2) patients with missing information on surgery and follow-up. Finally, a total of 2,925 participants were included in this study. The process of selecting participants was illustrated in Fig 1. The requirement of ethical approval for this was waived by the Institutional Review Board of General Hospital of Northern Theater Command, because the data was accessed from SEER (a publicly available database). The need for written informed consent was waived by the Institutional Review Board of General Hospital of Northern Theater Command due to retrospective nature of the study. All methods were performed in accordance with the relevant guidelines and regulations.

### Data collection

The primary endpoint of this study was considered as the OS and CSS among ovarian cancer patients with liver metastases. OS was defined as the time from diagnosis to death from any cause, while CSS was defined as the time from the date of diagnosis to death from liver metastasis of ovarian cancer [14]. The following variables were extracted from the SEER database: age, race/ethnicity, marital status, income, grade (I, II, III, IV or unknown), tumor size, local lymph node metastasis, histologic (carcinosarcoma, clear cell, endometrioid, malignant brenner carcinoma, mucinous, serous and other), combined bone metastasis, combined brain metastasis, combined lung metastasis, combined other sites metastasis, cancer antigen-125 (CA-125), tumor location (only one side, bilateral), residual tumor volume, surgery, radiotherapy, chemotherapy, treatment (receiving surgery and radiotherapy, only receiving surgery, only receiving radiotherapy, and none).

### Statistical analysis

Continuous variables were presented as mean ± standard deviation (Mean ± SD, normal distribution) or median and quartile range [M (Q1, Q3), non-normal distribution]. Student’s t-test or Mann-Whitney U test was used for between-group comparisons. The categorical variable was reported as the number of cases and composition ratio n (%), and group differences were compared using the chi-square test. *P*-value of less than 0.05 was deemed statistically significant.

We conducted a trend analysis of the incidence, OS and CSS among ovarian cancer patients with liver metastases in the SEER database from 2010 to 2019. Univariate Cox proportional risk model was utilized to identify potential covariates associated with OS (*P*<0.05), while the univariate competing risk model was employed to identify potential covariates associated with CSS (*P*<0.05). Subsequently, OS served as the outcome variable and different treatment methods were considered as independent variables, two models were established to investigate the association between different treatment methods and OS in ovarian cancer patients with liver metastases. Model 1 was univariate Cox proportional risk model (unadjusted covariates). Model 2 was multivariate Cox proportional risk model (adjusted all potential covariates associated with OS). Similarly, to assess the impact of various treatment methods on CSS, we developed univariate and multivariate competing risk models with CSS as the outcome and different treatment methods as independent variables. Model 1 was univariate competing risk model (unadjusted covariates). Model 2 was multivariate competing risk model (adjusted all potential covariates associated with CSS). The OS and CSS were calculated using the Kaplan-Meier curve. Subgroup analyses were conducted based on patients with different distant metastases types. In addition, we also developed online nomograms to predict patients’ OS and CSS, respectively. The concordance index (C-index) was calculated to verify the predicting performance of two nomogram. The statistical analysis was conducted utilizing SAS 9.4 software (SAS Institute Inc., Cary, NC, USA). R software was utilized for the computation of the average annual percent change (AAPC) and its corresponding 95% confidence interval (CI).

## Results

### Patients’ characteristics

A total of 2,925 ovarian cancer patients with liver metastases, with a mean age of 65.16 ± 13.49 years, were enrolled in this study. Most patients were white (78.46%). The median follow-up duration was 8.00 (1.00, 25.50) months, and at the conclusion of the follow-up period, a total of 689 patients remained alive. The OS and CSS rates were 76.44% and 72.99% for all patients, respectively. Detailed demographic and clinicopathological characteristics of all included patients were shown in Table 1.

**Table 1:** Demographic and clinicopathological characteristics of all included patients.

Variables	All patients (n = 2925)	Alive (n = 689)	Dead (n = 2236)	*P*
Age, year, Mean ± SD	65.16 ± 13.49	60.84 ± 12.76	66.49 ± 13.43	<0.001
Race/ethnicity, n (%)				<0.001
Black	346 (11.83)	57 (8.27)	289 (12.92)	
White	2295 (78.46)	532 (77.21)	1763 (78.85)	
Other	284 (9.71)	100 (14.51)	184 (8.23)	
Marital status, n (%)				<0.001
Married	1254 (42.87)	354 (51.38)	900 (40.25)	
Not married	1545 (52.82)	301 (43.69)	1244 (55.64)	
Unknown	126 (4.31)	34 (4.93)	92 (4.11)	
Income, n (%)				<0.001
<$70,000	1859 (63.56)	366 (53.12)	1493 (66.77)	
≥ $70,000	1066 (36.44)	323 (46.88)	743 (33.23)	
Grade, n (%)				0.391
Grade I & Grade II	97 (3.32)	28 (4.06)	69 (3.09)	
Grade III & Grade IV	889 (30.39)	202 (29.32)	687 (30.72)	
Unknown	1939 (66.29)	459 (66.62)	1480 (66.19)	
Tumor size, n (%)				<0.001
≤50	375 (12.82)	114 (16.55)	261 (11.67)	
50–100	561 (19.18)	166 (24.09)	395 (17.67)	
100–200	573 (19.59)	154 (22.35)	419 (18.74)	
>200	80 (2.74)	17 (2.47)	63 (2.82)	
Unknown	1336 (45.68)	238 (34.54)	1098 (49.11)	
Local lymph node metastasis, n (%)				<0.001
No	758 (25.91)	121 (17.56)	637 (28.49)	
Yes	566 (19.35)	74 (10.74)	492 (22.00)	
Unknown	1601 (54.74)	494 (71.70)	1107 (49.51)	
Histologic, n (%)				<0.001
Carcinosarcoma	111 (3.79)	22 (3.19)	89 (3.98)	
Clear cell	76 (2.60)	20 (2.90)	56 (2.50)	
Endometrioid	50 (1.71)	16 (2.32)	34 (1.52)	
Malignant Brenner Carcinoma	931 (31.83)	99 (14.37)	832 (37.21)	
Mucinous	69 (2.36)	5 (0.73)	64 (2.86)	
Serous	1325 (45.30)	475 (68.94)	850 (38.01)	
Other	363 (12.41)	52 (7.55)	311 (13.91)	
Combined bone metastasis, n (%)				<0.001
No	2632 (89.98)	661 (95.94)	1971 (88.15)	
Yes	179 (6.12)	16 (2.32)	163 (7.29)	
Unknown	114 (3.90)	12 (1.74)	102 (4.56)	
Combined brain metastasis, n (%)				<0.001
No	2776 (94.91)	675 (97.97)	2101 (93.96)	
Yes	29 (0.99)	1 (0.15)	28 (1.25)	
Unknown	120 (4.10)	13 (1.89)	107 (4.79)	
Combined lung metastasis, n (%)				<0.001
No	2081 (71.15)	568 (82.44)	1513 (67.67)	
Yes	709 (24.24)	105 (15.24)	604 (27.01)	
Unknown	135 (4.62)	16 (2.32)	119 (5.32)	
Combined other sites metastasis, n (%)				<0.001
No	2182 (74.60)	511 (74.17)	1671 (74.73)	
Yes	513 (17.54)	151 (21.92)	362 (16.19)	
Unknown	230 (7.86)	27 (3.92)	203 (9.08)	
CA-125, n (%)				<0.001
Negative/normal/within normal limits	73 (2.50)	21 (3.05)	52 (2.33)	
Positive/elevated	2246 (76.79)	577 (83.74)	1669 (74.64)	
Unknown	606 (20.72)	91 (13.21)	515 (23.03)	
Tumor location, n (%)				0.149
Only one side	1049 (35.86)	263 (38.17)	786 (35.15)	
Bilateral	1876 (64.14)	426 (61.83)	1450 (64.85)	
Residual tumor volume, n (%)				<0.001
No gross residual tumor nodules	350 (11.97)	190 (27.58)	160 (7.16)	
No cytoreductive surgery	1462 (49.98)	149 (21.63)	1313 (58.72)	
Optimal debulking	269 (9.20)	91 (13.21)	178 (7.96)	
Residual tumor nodule(s) greater than 1 cm	157 (5.37)	58 (8.42)	99 (4.43)	
Macroscopic residual tumor nodule(s), size not stated	159 (5.44)	36 (5.22)	123 (5.50)	
Unknown	528 (18.05)	165 (23.95)	363 (16.23)	
Surgery, n (%)				<0.001
Debulking	885 (30.26)	375 (54.43)	510 (22.81)	
Oophorectomy	147 (5.03)	56 (8.13)	91 (4.07)	
Oophorectomy with omentectomy	221 (7.56)	76 (11.03)	145 (6.48)	
Pelvic exenteration	55 (1.88)	26 (3.77)	29 (1.30)	
Natural orifice surgery	17 (0.58)	2 (0.29)	15 (0.67)	
None	1600 (54.70)	154 (22.35)	1446 (64.67)	
Radiotherapy, n (%)				0.018
Yes	69 (2.36)	8 (1.16)	61 (2.73)	
None	2856 (97.64)	681 (98.84)	2175 (97.27)	
Chemotherapy, n (%)				<0.001
Yes	1981 (67.73)	631 (91.58)	1350 (60.38)	
No/Unknown	944 (32.27)	58 (8.42)	886 (39.62)	
Treatment, n (%)				<0.001
None	1557 (53.23)	149 (21.63)	1408 (62.97)	
Surgery and radiotherapy	30 (1.03)	3 (0.44)	27 (1.21)	
Only receiving surgery	1299 (44.41)	532 (77.21)	767 (34.30)	
Only receiving radiotherapy	39 (1.33)	5 (0.73)	34 (1.52)	
Time, month, M (Q_1_, Q_3_)	8.00 (1.00, 25.00)	23.00 (8.00, 52.00)	5.00 (1.00, 19.00)	<0.001
Status, n (%)				<0.001
Alive	689 (23.56)	689 (100.00)	0 (0.00)	
Dead of liver metastasis of ovarian cancer	2135 (72.99)	0 (0.00)	2135 (95.48)	
Dead of other cause	101 (3.45)	0 (0.00)	101 (4.52)	

Abbreviations: CA-125 = cancer antigen-125; SD = standard deviation; M (Q1, Q3) = median and quartile range.

### The incidence and survival trends of liver metastases in ovarian cancer

**Fig 2:**
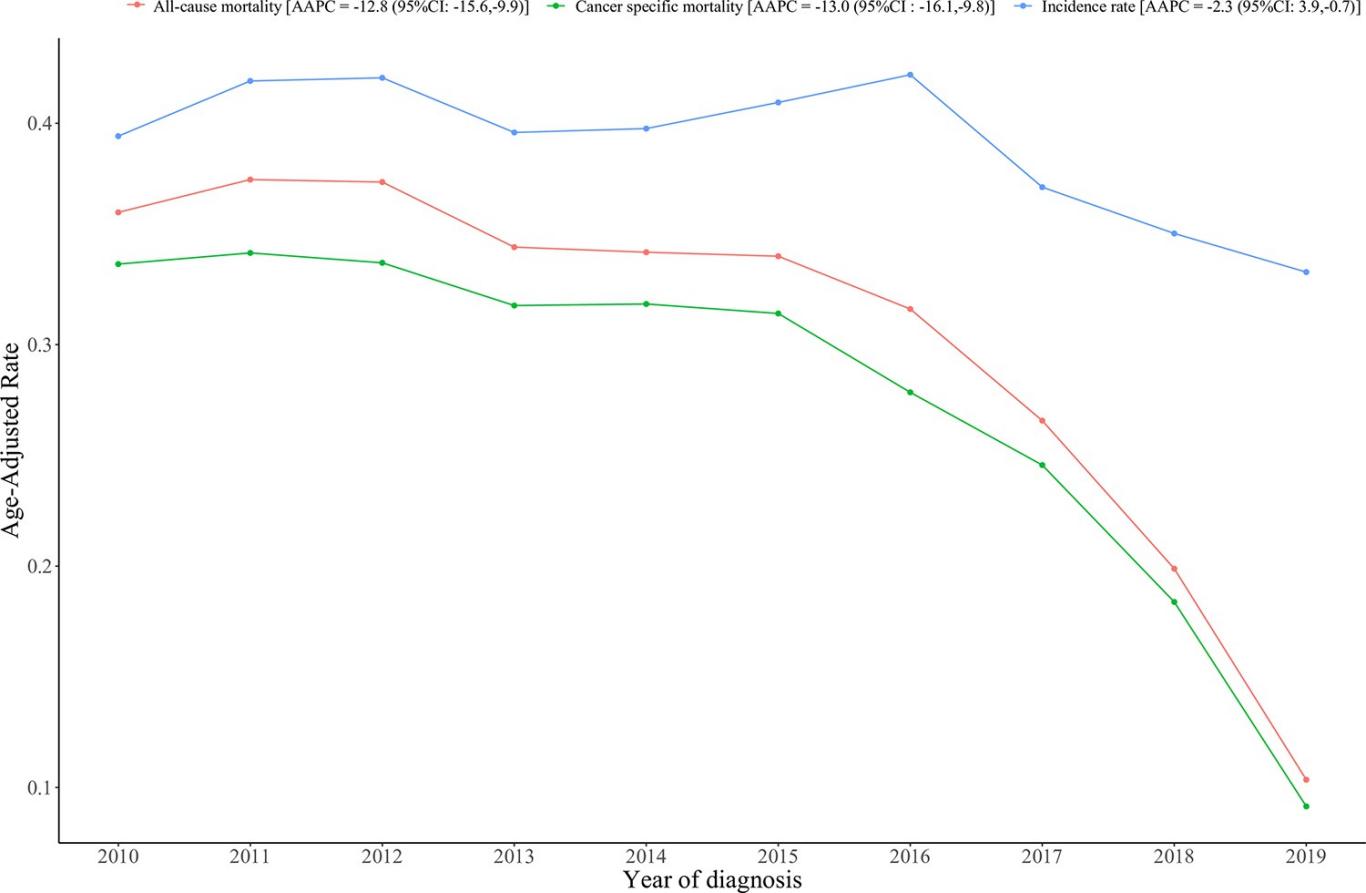
The incidence rates and mortality of liver metastases in ovarian cancer between 2010 and 2019.

Fig 2 illustrates trend in the incidence and mortality of liver metastases in ovarian cancer. The overall age-adjusted incidence rate of liver metastases in ovarian cancer was decreasing trend with an AAPC value of -2.3 (95%CI: -3.9, -0.7). Simultaneously, we conducted an analysis of the trends in OS and CSS among ovarian cancer patients with liver metastases. All-cause mortality and liver metastasis of ovarian cancer-specific mortality trends were declined, with AAPC value of -12.8 (95% CI: -15.6, -9.9) and -13.0 (95% CI: -16.1, -9.8), respectively. In addition, S1 Fig. also shows the incidence trend of liver metastases in ovarian cancer stratified by age, tumor grade, and treatment modality. Although there was no statistical significance in certain subgroups, all incidence rate trend showed a tendency to a decrease in liver metastases in ovarian cancer stratified by age, tumor grade, and treatment modality. S2 Fig. also demonstrates a declining trend in the all-cause mortality of liver metastases in ovarian cancer stratified by age, tumor grade, and treatment modality. Similar results were observed for specific mortality of liver metastases in ovarian cancer in different subgroups (S3 Fig).

### Impact of different treatments on survival of liver metastases in ovarian cancer

Univariate Cox proportional risk and competing risk analyses demonstrated that age, race/ethnicity, marital status, income, grade, tumor size, local lymph node metastasis, histologic, combined bone metastasis, combined brain metastasis, combined lung metastasis, combined other sites metastasis, CA-125, residual tumor volume may be covariates associated with OS and CSS (*P*<0.05) (S1 Table). As shown in Table 2, after adjusting all covariates, ovarian cancer patients with liver metastases who only received surgery was associated with OS [Model 2: hazard ratio (HR) = 0.39, 95%CI: 0.31–0.48, *P*<0.001]. Using debulking as a reference, the absence of surgery was identified as a significant risk factor for OS (Model 1: HR = 4.23, 95%CI: 3.80–4.70, *P*<0.001; Model 2: HR = 2.66, 95%CI: 2.13–3.33, *P*<0.001), while the impact of other surgical methods on OS was not statistically significant (*P*>0.05). Also, chemotherapy was found to be a significant protective factor for OS of ovarian cancer patients with liver metastases, even after adjusting for variables (Model 2: HR = 0.33, 95%CI: 0.30–0.37, *P*<0.001). Similarly, after adjusting all covariates, in terms of CSS of ovarian cancer patients with liver metastases, only receiving surgery (Model 2: HR = 0.37, 95%CI: 0.30–0.47, *P*<0.001) and chemotherapy (Model 2: HR = 0.44, 95%CI: 0.39–0.50, *P*<0.001) were significant protective factor, while not receiving surgery (Model 2: HR = 2.68, 95%CI: 2.10–3.43, *P*<0.001) remained a risk factor. The Kaplan-Meier curve showed that only received surgery had a higher OS (S4A Fig) and CSS (S5A Fig). Surgical method also appeared to affect patient survival, with pelvic exenteration were found to have a higher OS (S4B Fig) and CSS than others (S5B Fig). Furthermore, patients who received radiotherapy may have lower OS (S4C Fig) and CSS (S5C Fig). The impact of chemotherapy on prognosis of patients with ovarian cancer was obvious, leading to significantly improved OS (S4D Fig) and CSS (S5D Fig) compared to those who do not receive this treatment.

**Table 2:** Association between different treatment methods and OS/ CSS in ovarian cancer patients with liver metastases.

Outcomes	Variables	Model 1		Model 2	
		HR (95%CI)	*P*	HR (95%CI)	*P*
OS	Treatment				
	None	Ref		Ref	
	Surgery and radiotherapy	0.72 (0.49–1.05)	0.088	0.88 (0.57–1.35)	0.545
	Only receiving surgery	0.24 (0.22–0.27)	<0.001	0.39 (0.31–0.48)	<0.001
	Only receiving radiotherapy	0.91 (0.65–1.28)	0.597	0.72 (0.51–1.03)	0.073
	Surgery				
	Debulking	Ref		Ref	
	Oophorectomy	1.19 (0.95–1.49)	0.125	1.12 (0.89–1.41)	0.347
	Oophorectomy with omentectomy	1.17 (0.97–1.40)	0.100	1.15 (0.95–1.40)	0.142
	Pelvic exenteration	0.78 (0.54–1.14)	0.199	0.81 (0.55–1.18)	0.267
	Natural orifice surgery	2.05 (1.23–3.43)	0.006	1.65 (0.98–2.80)	0.061
	None	4.23 (3.80–4.70)	<0.001	2.66 (2.13–3.33)	<0.001
	Radiotherapy				
	None	Ref		Ref	
	Yes	1.53 (1.19–1.97)	0.001	1.07 (0.82–1.41)	0.613
	Chemotherapy				
	No/Unknown	Ref		Ref	
	Yes	0.22 (0.21–0.25)	<0.001	0.33 (0.30–0.37)	<0.001
CSS	Treatment				
	None	Ref		Ref	
	Surgery and radiotherapy	0.76 (0.59–0.99)	0.046	0.71 (0.49–1.04)	0.077
	Only receiving surgery	0.30 (0.27–0.33)	<0.001	0.37 (0.30–0.47)	<0.001
	Only receiving radiotherapy	1.08 (0.80–1.45)	0.628	0.85 (0.63–1.14)	0.282
	Surgery				
	Debulking	Ref		Ref	
	Oophorectomy	1.20 (0.98–1.48)	0.081	1.15 (0.94–1.40)	0.180
	Oophorectomy with omentectomy	1.14 (0.97–1.35)	0.115	1.13 (0.96–1.33)	0.156
	Pelvic exenteration	0.81 (0.58–1.12)	0.200	0.82 (0.60–1.14)	0.247
	Natural orifice surgery	1.81 (1.16–2.81)	0.009	1.61 (0.99–2.63)	0.055
	None	3.42 (3.10–3.77)	<0.001	2.68 (2.10–3.43)	<0.001
	Radiotherapy				
	None	Ref		Ref	
	Yes	1.57 (1.31–1.90)	<0.001	1.11 (0.84–1.46)	0.474
	Chemotherapy				
	No/Unknown	Ref		Ref	
	Yes	0.31 (0.28–0.34)	<0.001	0.44 (0.39–0.50)	<0.001

Abbreviations: OS = overall survival; CSS = cancer-specific survival; HR = hazard ratio; CI = confidence interval; Ref = reference. Model 1: crude model; Model 2: adjusted age, race/ethnicity, marital status, income, grade, tumor size, local lymph node metastasis, histologic, combined bone metastasis, combined brain metastasis, combined lung metastasis, combined other sites metastasis, cancer antigen-125, residual tumor volume

### Subgroup analyses based on patients with different distant metastases types

In order to better explore the effects of treatment modalities on OS and CSS in ovarian cancer patients with liver metastases who had different characteristics, we conducted a stratified analysis based on different distant metastases types. As shown in Table 3, we found that only receiving surgery and chemotherapy still were significant protective factor of OS and CSS for patients without other distant metastases (Subgroup Ⅰ), with distant metastases to the bone, lung, brain or other organs (Subgroup Ⅱ), with bone metastasis (Subgroup Ⅲ), and with lung metastasis (Subgroup Ⅳ). Additionally, for ovarian cancer patients with liver metastases who combined bone metastasis, surgery type, including oophorectomy with omentectomy (HR = 0.42, 95%CI: 0.21–0.84, *P* = 0.014), pelvic exenteration (HR = 0.24, 95%CI: 0.06–0.98, *P* = 0.047) and natural orifice surgery (HR = 3.15, 95%CI: 1.02–9.69, *P* = 0.046) was considered to be associated with CSS.

**Table 3:** Subgroup analyses based on patients with different distant metastases types.

Population	Variables	OS		CSS	
		HR (95%CI)	*P*	HR (95%CI)	*P*
Subgroup Ⅰ: Patients without other sites metastasis (n = 1556)	Treatment				
	None	Ref		Ref	
	Surgery and radiotherapy	0.93 (0.42–2.07)	0.860	0.98 (0.61–1.57)	0.923
	Only receiving surgery	0.34 (0.25–0.47)	<0.001	0.39 (0.27–0.56)	<0.001
	Only receiving radiotherapy	0.38 (0.14–1.04)	0.060	0.50 (0.15–1.65)	0.256
	Surgery				
	Debulking	Ref		Ref	
	None	2.96 (2.17–4.05)	<0.001	2.67 (1.83–3.88)	<0.001
	Oophorectomy	1.03 (0.75–1.41)	0.860	1.05 (0.80–1.39)	0.725
	Oophorectomy with omentectomy	1.14 (0.89–1.46)	0.311	1.15 (0.93–1.44)	0.201
	Pelvic exenteration	0.77 (0.46–1.29)	0.326	0.77 (0.49–1.20)	0.244
	Natural orifice surgery	1.68 (0.85–3.34)	0.136	1.54 (0.80–2.96)	0.201
	Radiotherapy				
	None	Ref		Ref	
	Yes	0.90 (0.49–1.64)	0.721	1.06 (0.46–2.43)	0.897
	Chemotherapy				
	No/Unknown	Ref		Ref	
	Yes	0.32 (0.27–0.37)	<0.001	0.42 (0.35–0.50)	<0.001
Subgroup Ⅱ: Patients combined bone metastasis, brain metastasis, lung metastasis, other sites metastasis (n = 1092)	Treatment				
	None	Ref		Ref	
	Surgery and radiotherapy	1.08 (0.60–1.93)	0.804	0.69 (0.40–1.18)	0.174
	Only receiving surgery	0.36 (0.25–0.53)	<0.001	0.29 (0.20–0.41)	<0.001
	Only receiving radiotherapy	1.10 (0.74–1.65)	0.634	1.23 (0.90–1.68)	0.187
	Surgery				
	Debulking	Ref		Ref	
	None	3.00 (1.98–4.55)	<0.001	3.43 (2.35–5.00)	<0.001
	Oophorectomy	1.13 (0.74–1.72)	0.581	1.12 (0.77–1.63)	0.563
	Oophorectomy with omentectomy	1.18 (0.84–1.67)	0.337	1.09 (0.80–1.49)	0.593
	Pelvic exenteration	0.77 (0.43–1.40)	0.392	0.81 (0.46–1.46)	0.489
	Natural orifice surgery	1.77 (0.63–4.96)	0.275	2.03 (0.73–5.62)	0.174
	Radiotherapy				
	None	Ref		Ref	
	Yes	1.55 (1.13–2.13)	0.007	1.48 (1.11–1.97)	0.007
	Chemotherapy				
	No/Unknown	Ref		Ref	
	Yes	0.29 (0.25–0.35)	<0.001	0.39 (0.32–0.47)	<0.001
Subgroup Ⅲ: Patients combined bone metastasis (n = 179)	Treatment				
	None	Ref		Ref	
	Surgery and radiotherapy	0.40 (0.13–1.22)	0.108	0.34 (0.15–0.81)	0.014
	Only receiving surgery	0.28 (0.11–0.72)	0.008	0.28 (0.13–0.58)	<0.001
	Only receiving radiotherapy	0.86 (0.45–1.62)	0.639	0.86 (0.51–1.45)	0.566
	Surgery				
	Debulking	Ref		Ref	
	None	3.17 (1.04–9.65)	0.042	2.21 (0.89–5.46)	0.087
	Oophorectomy	2.79 (0.73–10.65)	0.134	3.01 (0.80–11.31)	0.102
	Oophorectomy with omentectomy	0.44 (0.15–1.29)	0.135	0.42 (0.21–0.84)	0.014
	Pelvic exenteration	0.51 (0.04–6.71)	0.612	0.24 (0.06–0.98)	0.047
	Natural orifice surgery	3.69 (0.37–37.28)	0.268	3.15 (1.02–9.69)	0.046
	Radiotherapy				
	None	Ref		Ref	
	Yes	0.92 (0.53–1.59)	0.770	0.87 (0.54–1.39)	0.555
	Chemotherapy				
	No/Unknown	Ref		Ref	
	Yes	0.43 (0.28–0.66)	<0.001	0.43 (0.29–0.66)	<0.001
Subgroup Ⅳ: Patients combined lung metastasis (n = 709)	Treatment				
	None	Ref		Ref	
	Surgery and radiotherapy	0.75 (0.36–1.56)	0.441	0.47 (0.25–0.90)	0.022
	Only receiving surgery	0.36 (0.22–0.58)	<0.001	0.32 (0.20–0.49)	<0.001
	Only receiving radiotherapy	0.85 (0.51–1.42)	0.536	0.97 (0.70–1.33)	0.838
	Surgery				
	Debulking	Ref		Ref	
	None	2.78 (1.65–4.70)	<0.001	2.91 (1.78–4.75)	<0.001
	Oophorectomy	1.06 (0.61–1.84)	0.839	1.11 (0.71–1.75)	0.639
	Oophorectomy with omentectomy	1.02 (0.66–1.57)	0.928	0.94 (0.64–1.40)	0.773
	Pelvic exenteration	1.10 (0.52–2.32)	0.808	1.12 (0.67–1.87)	0.674
	Natural orifice surgery	1.31 (0.39–4.36)	0.657	1.42 (0.41–4.92)	0.580
	Radiotherapy				
	None	Ref		Ref	
	Yes	1.18 (0.77–1.79)	0.453	1.09 (0.77–1.53)	0.637
	Chemotherapy				
	No/Unknown	Ref		Ref	
	Yes	0.27 (0.22–0.33)	<0.001	0.39 (0.32–0.49)	<0.001

Abbreviations: OS = overall survival; CSS = cancer-specific survival; HR = hazard ratio; CI = confidence interval; Ref = reference. Adjusted age, race/ethnicity, marital status, income, grade, tumor size, local lymph node metastasis, histologic, combined bone metastasis (not was adjusted in Subgroup Ⅱ and Ⅲ), combined brain metastasis (not was adjusted in Subgroup Ⅱ), combined lung metastasis (not was adjusted in Subgroup Ⅱ and Ⅳ), combined other sites metastasis (not was adjusted in Subgroup Ⅱ), cancer antigen-125, residual tumor volume.

### Establishment and validation of the nomogram

In univariate Cox proportional risk and competing risk analyses, age, race/ethnicity, marital status, income, grade, tumor size, local lymph node metastasis, histologic, combined bone metastasis, combined brain metastasis, combined lung metastasis, combined other sites metastasis, CA-125, and residual tumor volume were associated with both OS and CSS (*P*<0.05) (S1 Table). Thus, online OS (https://dynamic-nomogram-for-predicting-overall-survival—2023.shinyapps.io/DynNomapp/) and CSS (https://dynamic-nomogram-for-predicting-cancer-specific-survival—2023.shinyapps.io/DynNomapp/) predicting nomogram were established, respectively. C-index was used to verify the predicting performance of two nomogram (Table 4). Specifically, C-index of OS nomogram was 0.756 (95% CI: 0.746–0.766), and OS nomogram was 0.752 (95%CI: 0.742–0.763). These findings also indicated that two online nomograms had a good predicting value for OS and CSS in patients with ovarian cancer liver metastases.

**Table 4:** C-indexes for the nomograms.

Nomogram	C-index (95%CI)
OS predicting nomogram	0.756 (0.746–0.766)
CSS predicting nomogram	0.752 (0.742–0.763)

## Discussion

It is crucial to explore diverse treatment options for patients with liver metastases from ovarian cancer in order to improve patient prognosis. In this study, utilizing data from the SEER 2010–2019 registry, we examined the trends in morbidity and mortality among patients with liver metastases from ovarian cancer. Additionally, only receiving surgery and chemotherapy were also found to be significant protective factor for OS and CSS of patients.

Liver metastases are a frequent occurrence in patients diagnosed with ovarian cancer and are widely recognized as the primary cause of mortality associated with this disease [15]. The findings of this study revealed a noteworthy decrease in the incidence of liver metastases among ovarian cancer patients in the United States between 2010 and 2019. Additionally, there was a significant decreasing trend in the all-cause mortality/ cancer-specific mortality between 2010 and 2019. This could be attributed to timely intervention, and advancements in therapeutic approaches [16]. Although there was no statistical significance in certain subgroups, all incidence rate, all-cause mortality, cancer specific mortality trend showed a tendency to a decrease in ovarian cancer with liver metastases when stratified by age, tumor grade, and treatment modality.

Distant metastatic sites have a significant impact on the OS of patients with ovarian cancer that has spread [5]. Treatment strategies may vary depending on the specific sites of metastasis. Numerous therapeutic approaches for ovarian cancer with different sites of metastasis are currently under extensive investigation. For example, a review has demonstrated that whole brain radiotherapy (WBRT) is a viable and efficacious treatment modality for ovarian cancer patients with brain metastases [17]. In the study conducted by Cao et al., it was reported that chemotherapy and surgery were associated with lung metastases from ovarian cancer [18], and there was no significant difference in radiotherapy between ovarian cancer patients with or without lung metastasis. Therapeutic approaches play a crucial role in guiding clinical practice [19, 20]; however, the prognostic impact of various therapeutic strategies on patients with ovarian cancer liver metastases remains unclear to date. In our research, we incorporated more clinicopathological information. After adjusting for all potential confounding factors, the findings revealed that only receiving surgery were associated with OS and CSS of patients. A study of 72 patients with brain metastases from ovarian cancer showed that the combination of surgery and WBRT (median survival time: 23.07 months) resulted in superior survival outcomes compared to either surgery alone (median survival time: 6.90 months) or WBRT alone (median survival time: 5.33 months) [21]. Our study revealed no significant difference in the correlation between surgery combined with radiotherapy and OS/CSS among ovarian cancer patients with liver metastasis. Furthermore, in all subgroup analyses, only receiving surgery and chemotherapy also were beneficial for OS/CSS for patients. Similar to the findings in the general population, we observed no significant impact of different surgical method on patients’ OS and CSS in subgroups without other distant metastases (Subgroup Ⅰ), with distant metastases to the bone, lung, brain or other organs (Subgroup Ⅱ), and with lung metastasis (Subgroup Ⅳ) (*P*>0.05). Notably, when comparing to Debulking as a reference, the absence of surgery posed a risk factor for both OS and CSS within these specific subgroups. This observation may be attributed to the limited sample size of our study. It is worth noting that oophorectomy with omentectomy, pelvic exenteration and natural orifice surgery were related to CSS for ovarian cancer patients with liver metastases who combined bone metastasis. These findings may also imply that the type of surgery has an impact on the prognosis of diverse patients. Further prospective studies with larger sample size should be conducted to confirm our findings.

Additionally, this study also proposed two online dynamic nomogram to predict OS and CSS among ovarian cancer patients with liver metastasis, respectively. Overall, two model exhibited a good prediction performance. In clinical practice, clinicians can utilize these user-friendly dynamic nomograms to make early intervention decisions, which may improve prognoses of patients.

There are several limitations to this study. Firstly, the SEER database solely documented the metastases of liver, brain, lung, bone and distant lymph nodes; hence the specific metastases of other sites such as peritoneal metastases remain unknown [22]. Secondly, variables pertaining to comorbidities, types of chemotherapy administered and adjuvant agents as well as the sequence of treatment were not extracted form SEER database [23]. Thirdly, due to the limited sample size, it was not possible to conduct subgroup analysis on patients with distant metastases to the liver and brain. Fourthly, as mentioned by Forte S et al., liver resection is feasible during either primary debulking surgery (PDS) or interval debulking surgery (IDS) [24]. But all the data were obtained from the SEER public database, which prevented us from distinguishing between the sequencing of patients’ chemotherapy and surgery, as well as differentiating between PDS and IDS patients. Lastly, because the patient cohort in this study was limited to the US population, further validation of these findings is necessary across diverse populations worldwide.

## Conclusion

In summary, our research has elucidated a downward trend in morbidity and mortality rates among patients with liver metastases originating from ovarian cancer. Only receiving surgery and chemotherapy were protective factor for OS and CSS of patients. The findings of this study provide valuable guidance for clinicians and patients in selecting optimal treatment modalities to enhance the prognosis of individuals with liver metastases from ovarian cancer.

## Supporting information

S1 Fig The incidence trend of liver metastases in ovarian cancer stratified by (a) age, (b) tumor grade, (c) surgery, (d) radiotherapy and (e) chemotherapy.
(PDF)

S2 Fig The all-cause mortality trend of liver metastases in ovarian cancer stratified by (a) age, (b) tumor grade, (c) surgery, (d) radiotherapy and (e) chemotherapy.
(PDF)

S3 Fig The specific mortality trend of liver metastases in ovarian cancer stratified by (a) age, (b) tumor grade, (c) surgery, (d) radiotherapy and (e) chemotherapy.
(PDF)

S4 Fig Kaplan-Meier curve of OS. (PDF)

S5 Fig Kaplan-Meier curve of CSS. (PDF)

S1 Table Screening of confounding factors. (DOCX)
