# Supplementary figures and images for: Effect of different treatment modalities on ovarian cancer patients with liver metastases: A retrospective cohort study based on SEER
Source: PLoS One. 2024 Apr 18;19(4):e0299504. doi: 10.1371/journal.pone.0299504 (PMC11025763; doi:10.1371/journal.pone.0299504)

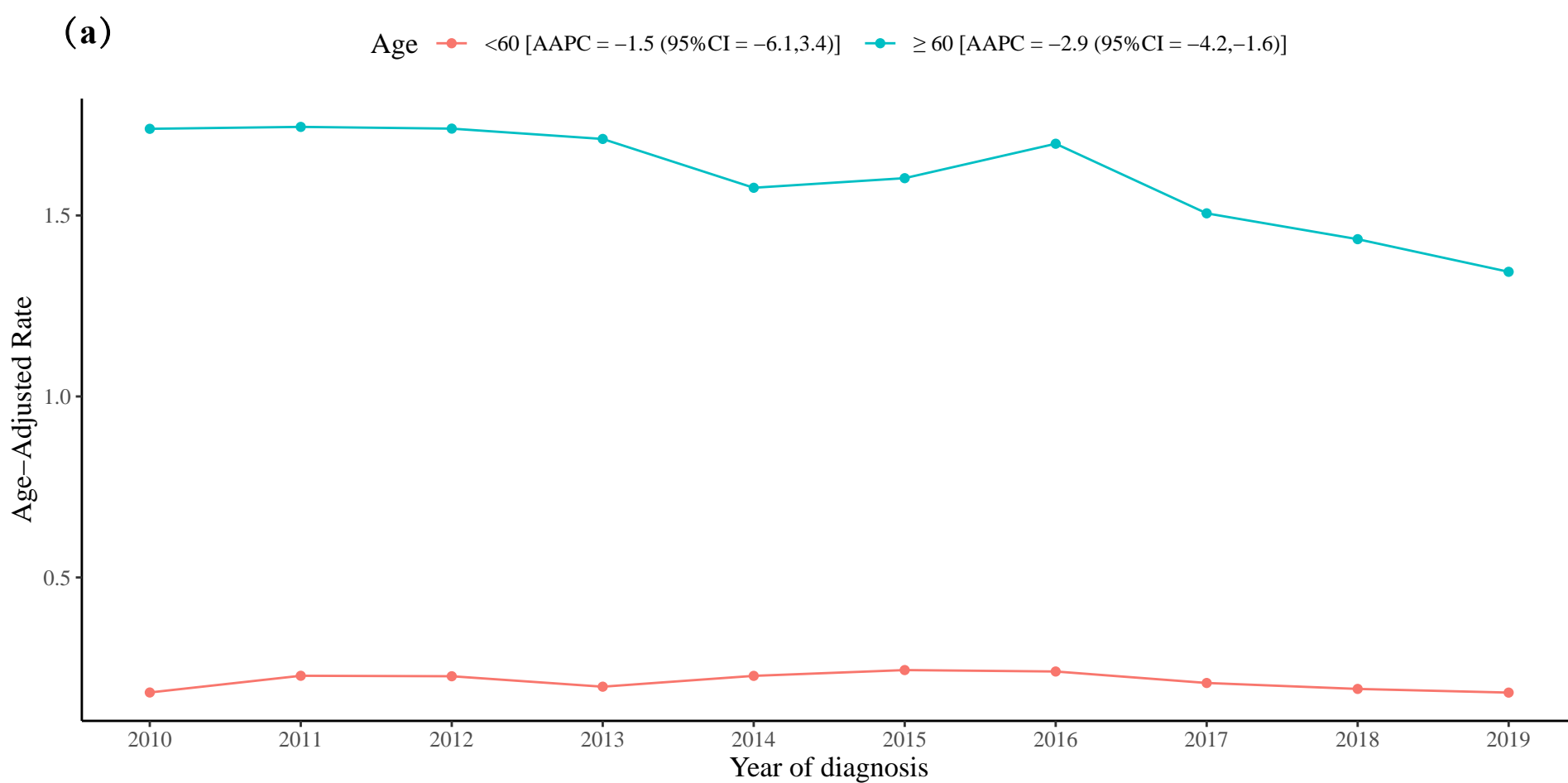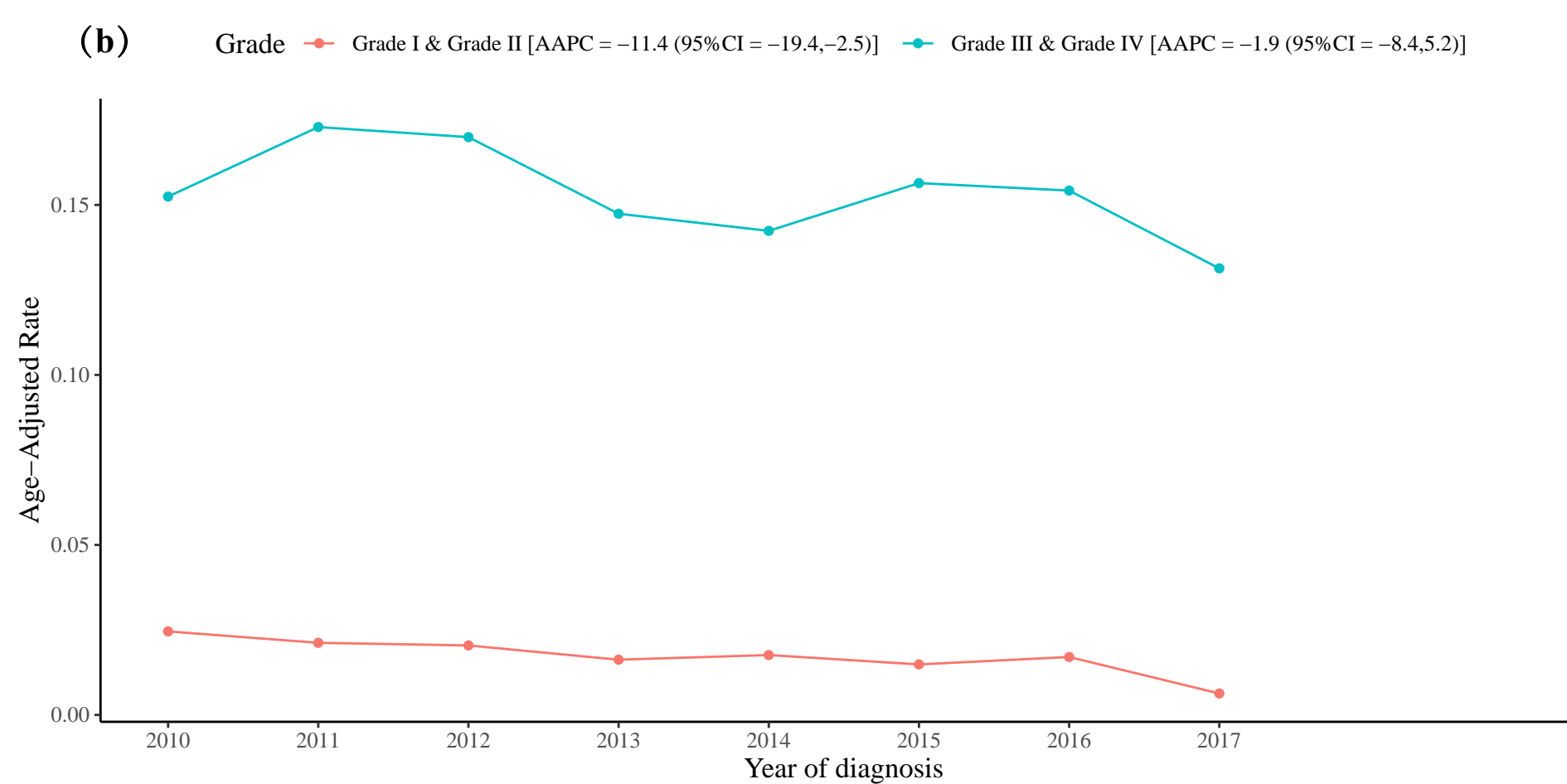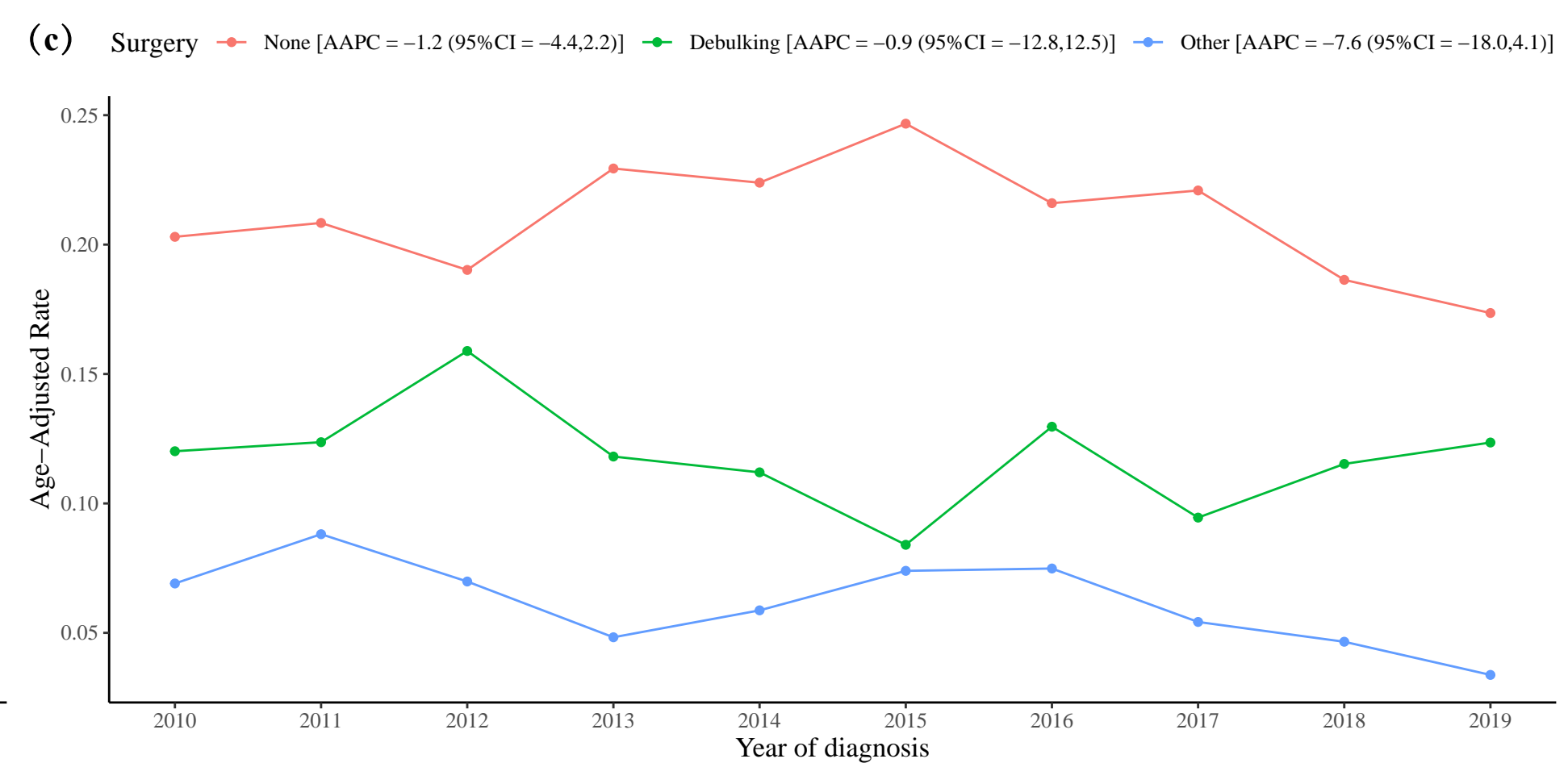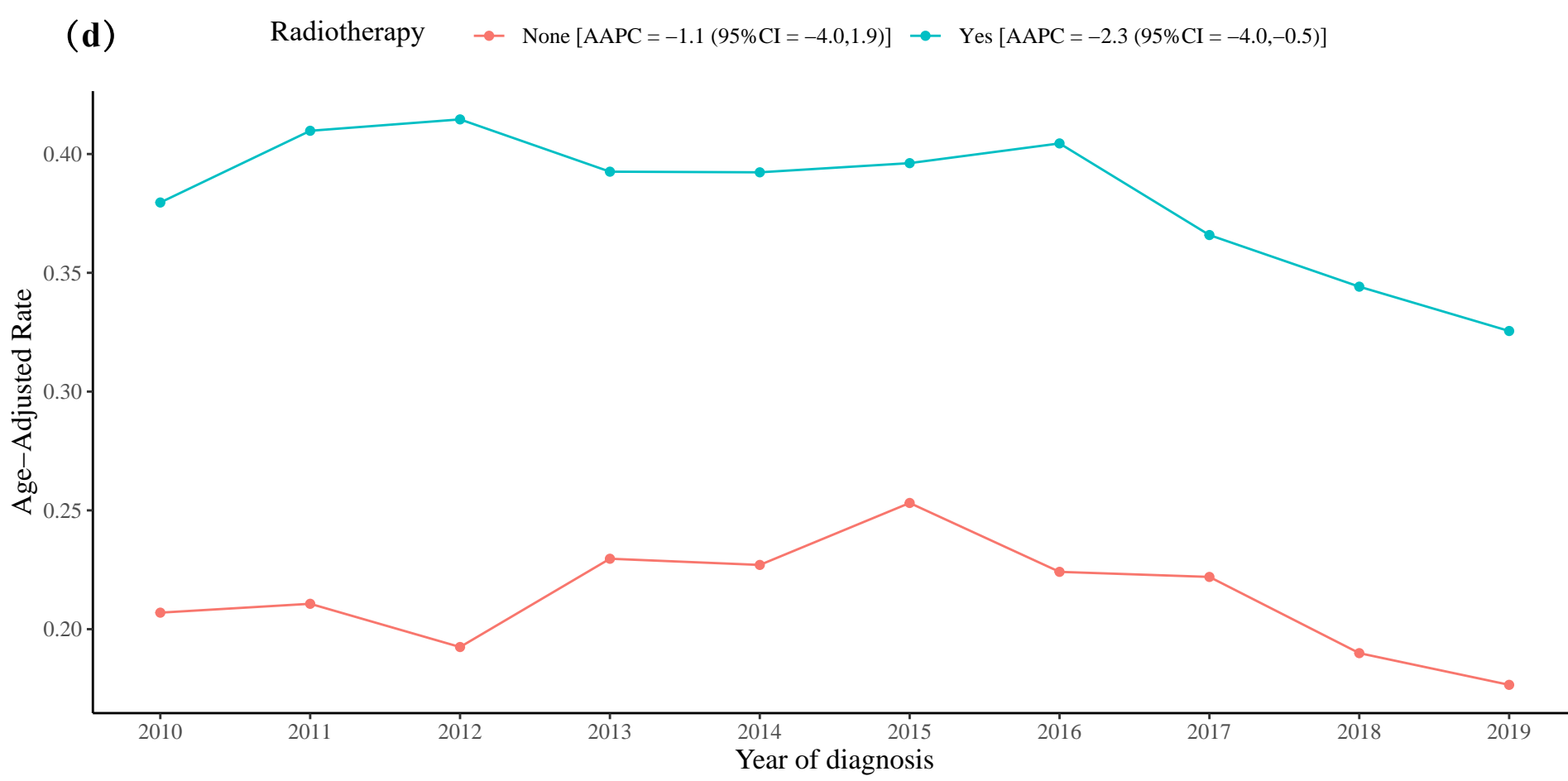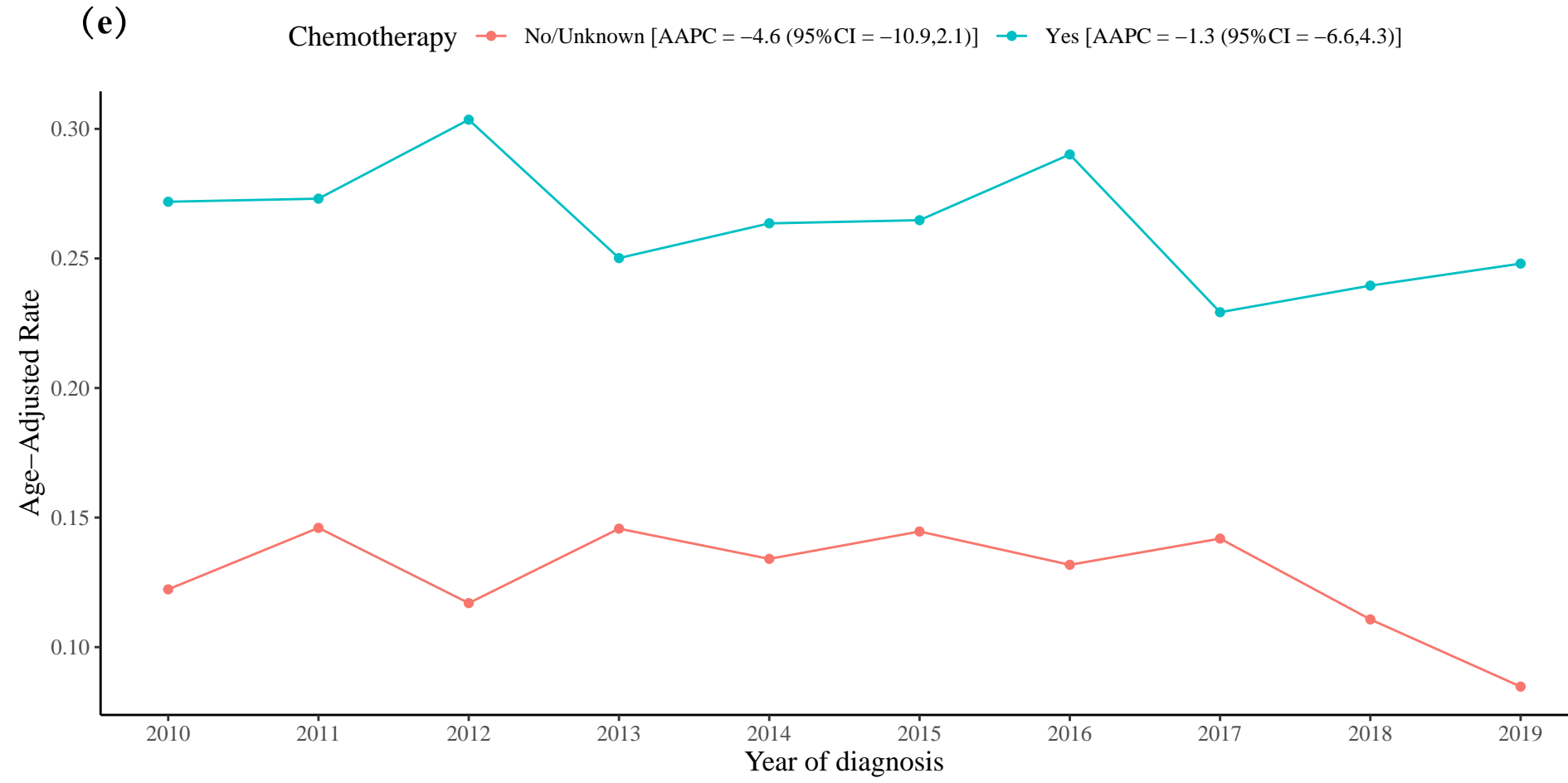

Supplement: S1 Fig — The incidence trend of liver metastases in ovarian cancer stratified by (a) age, (b) tumor grade, (c) surgery, (d) radiotherapy and (e) chemotherapy. (PDF) [file pone.0299504.s001.pdf]

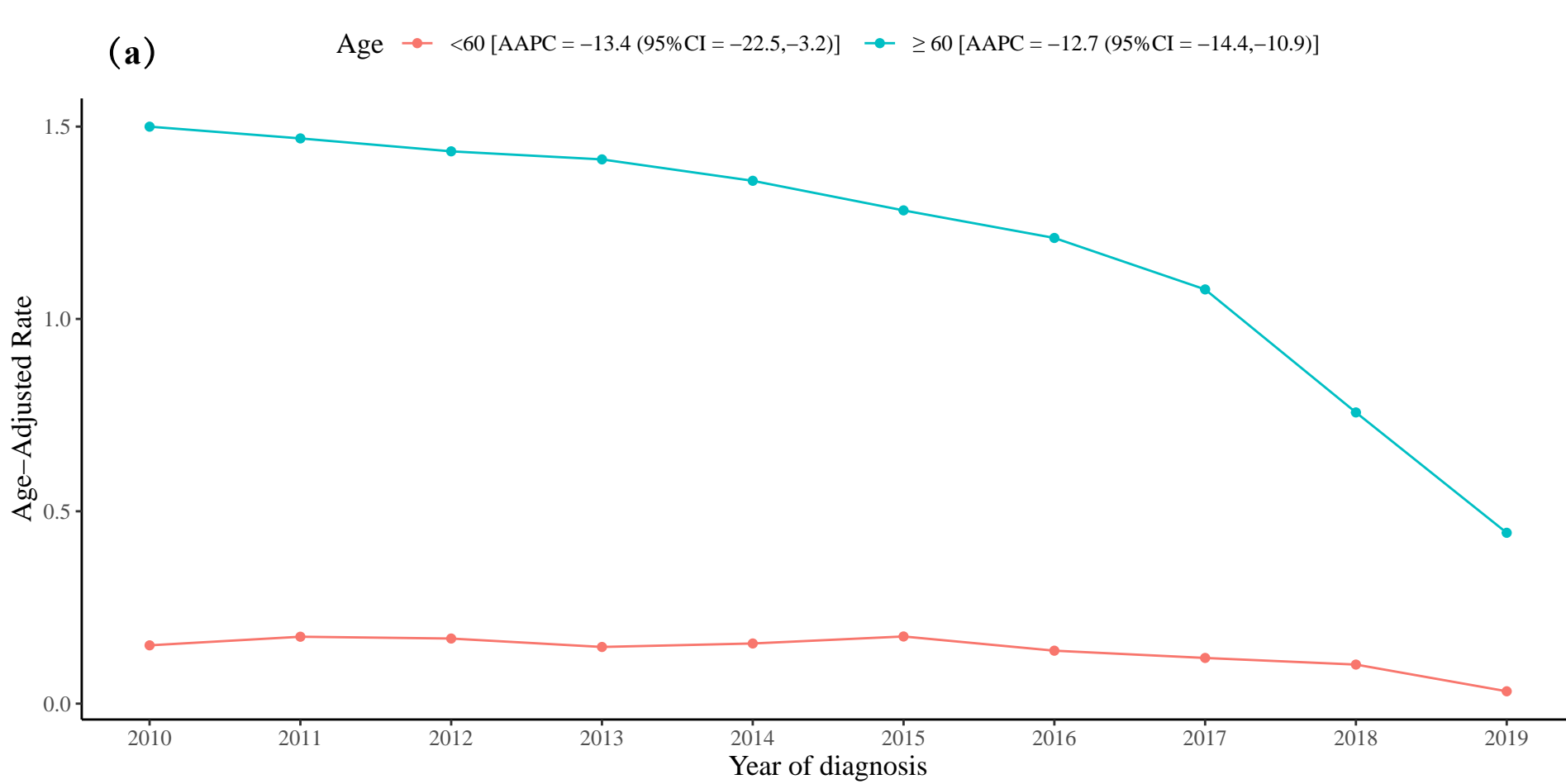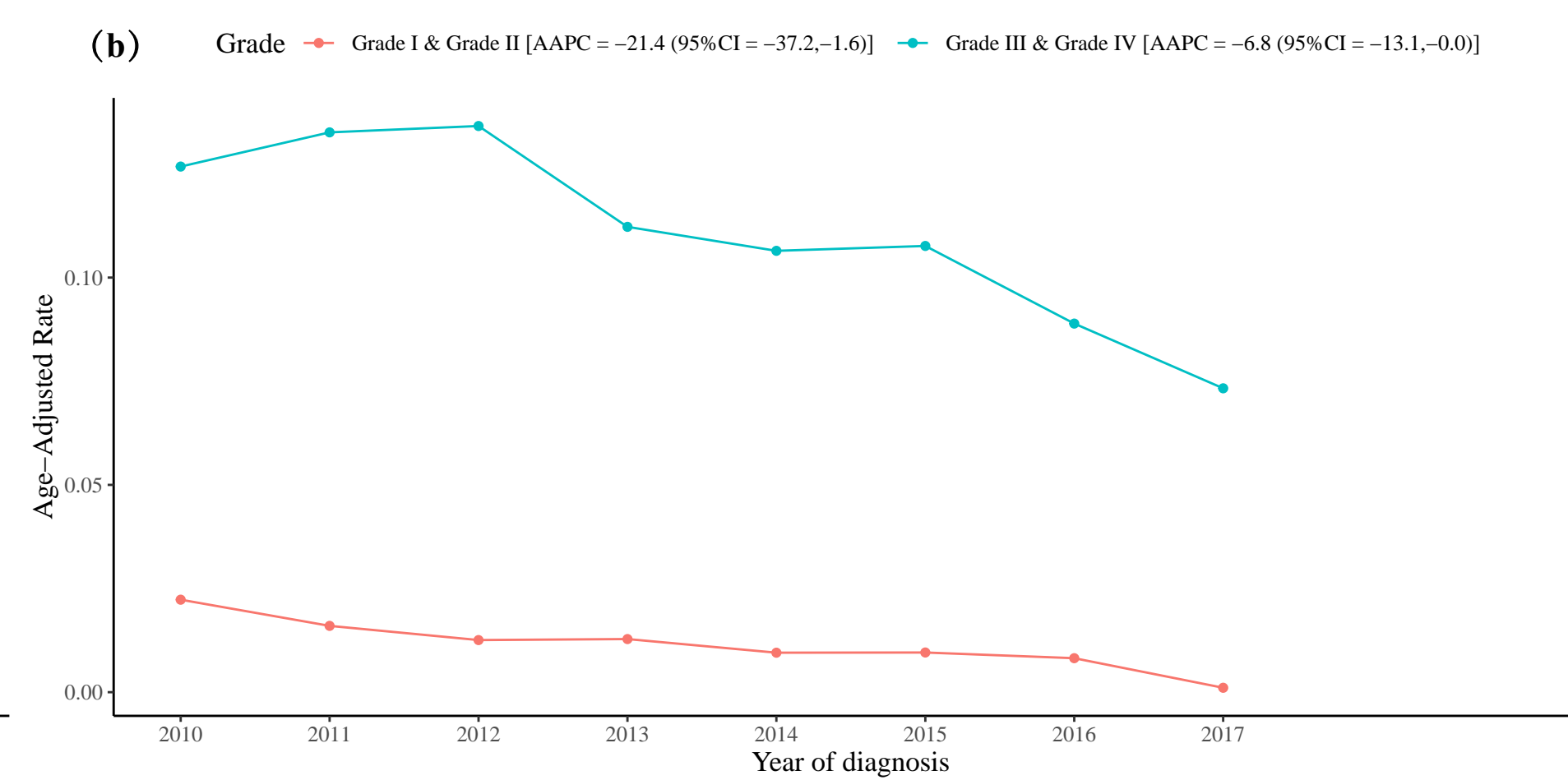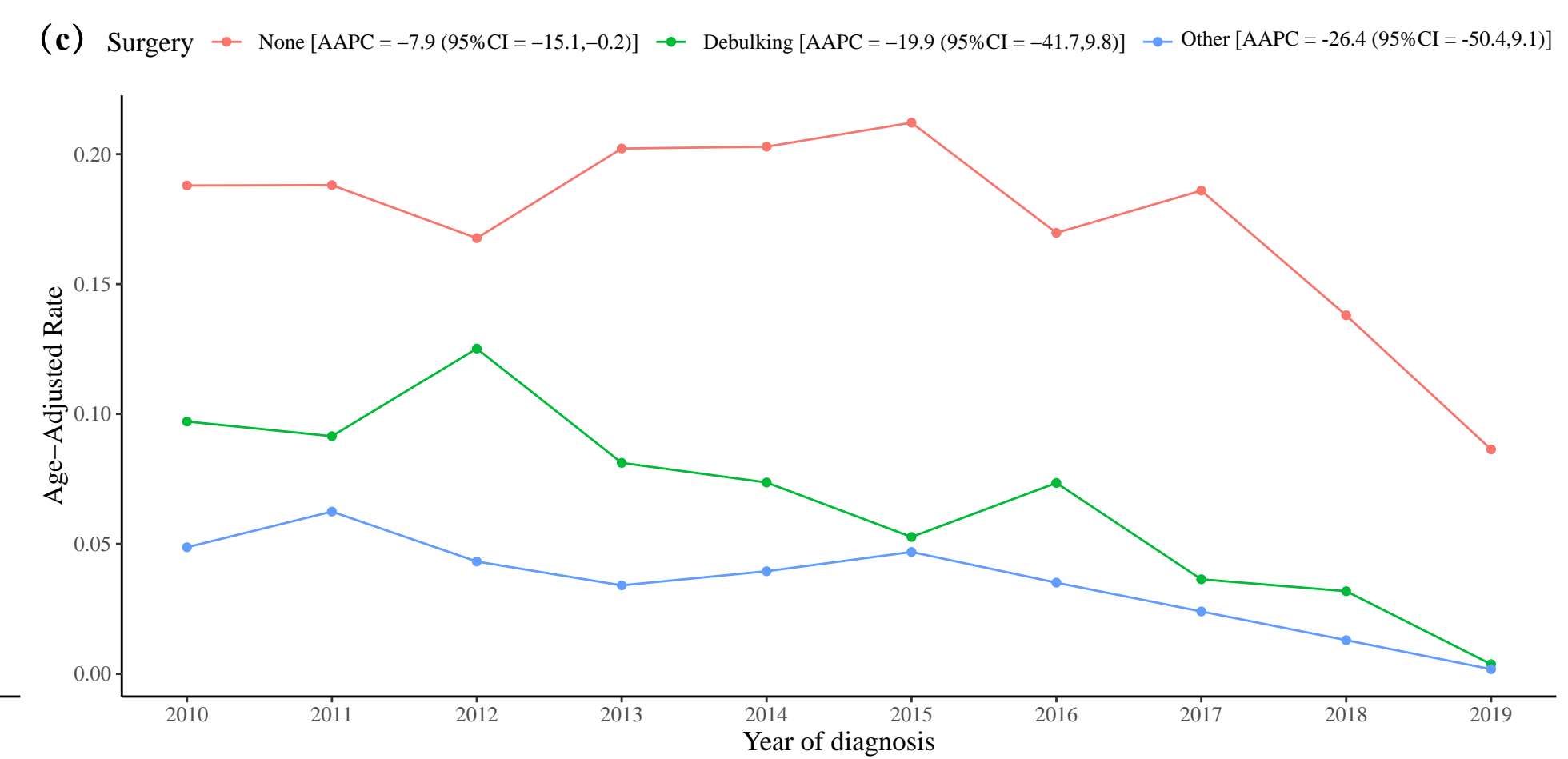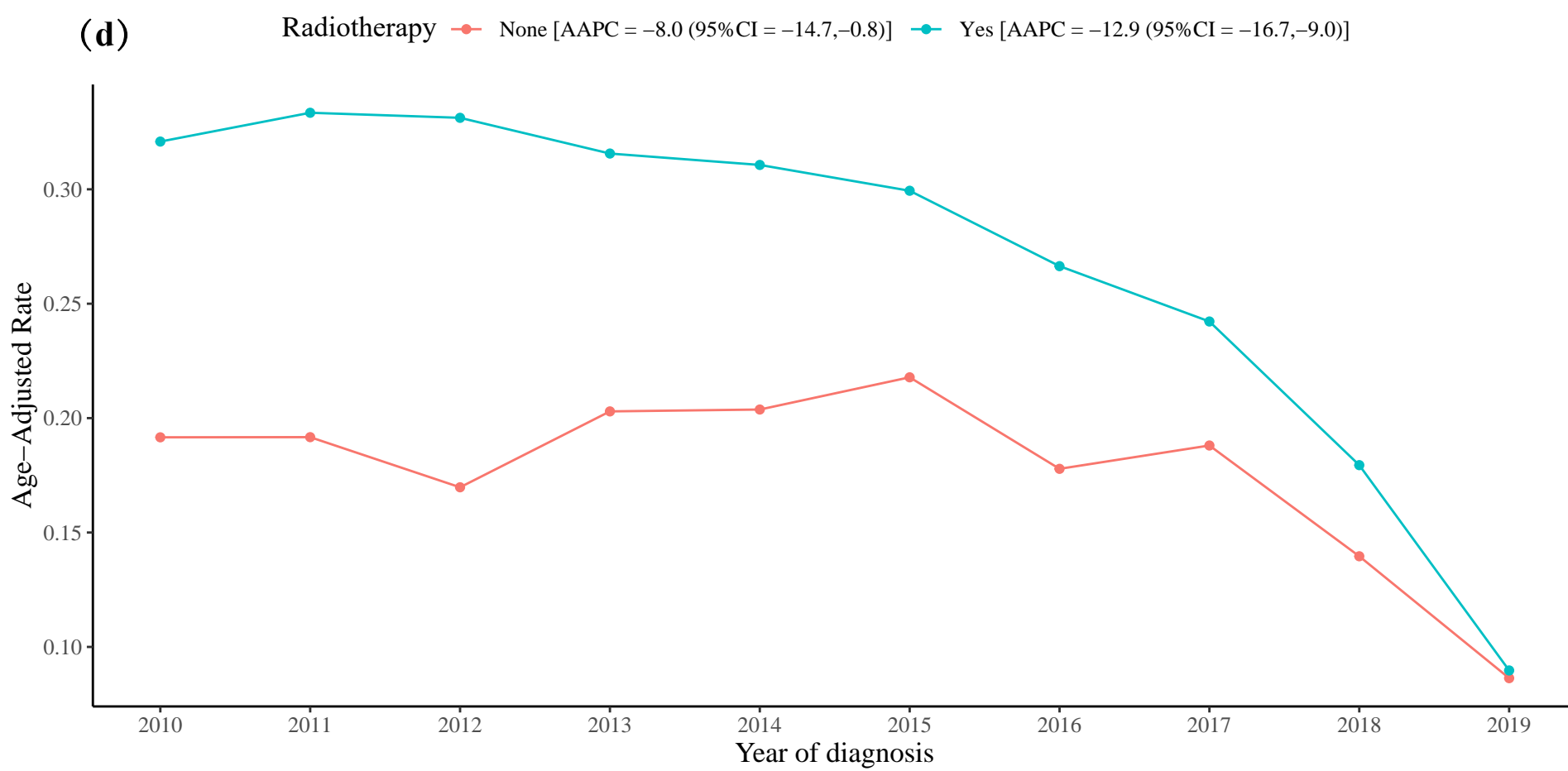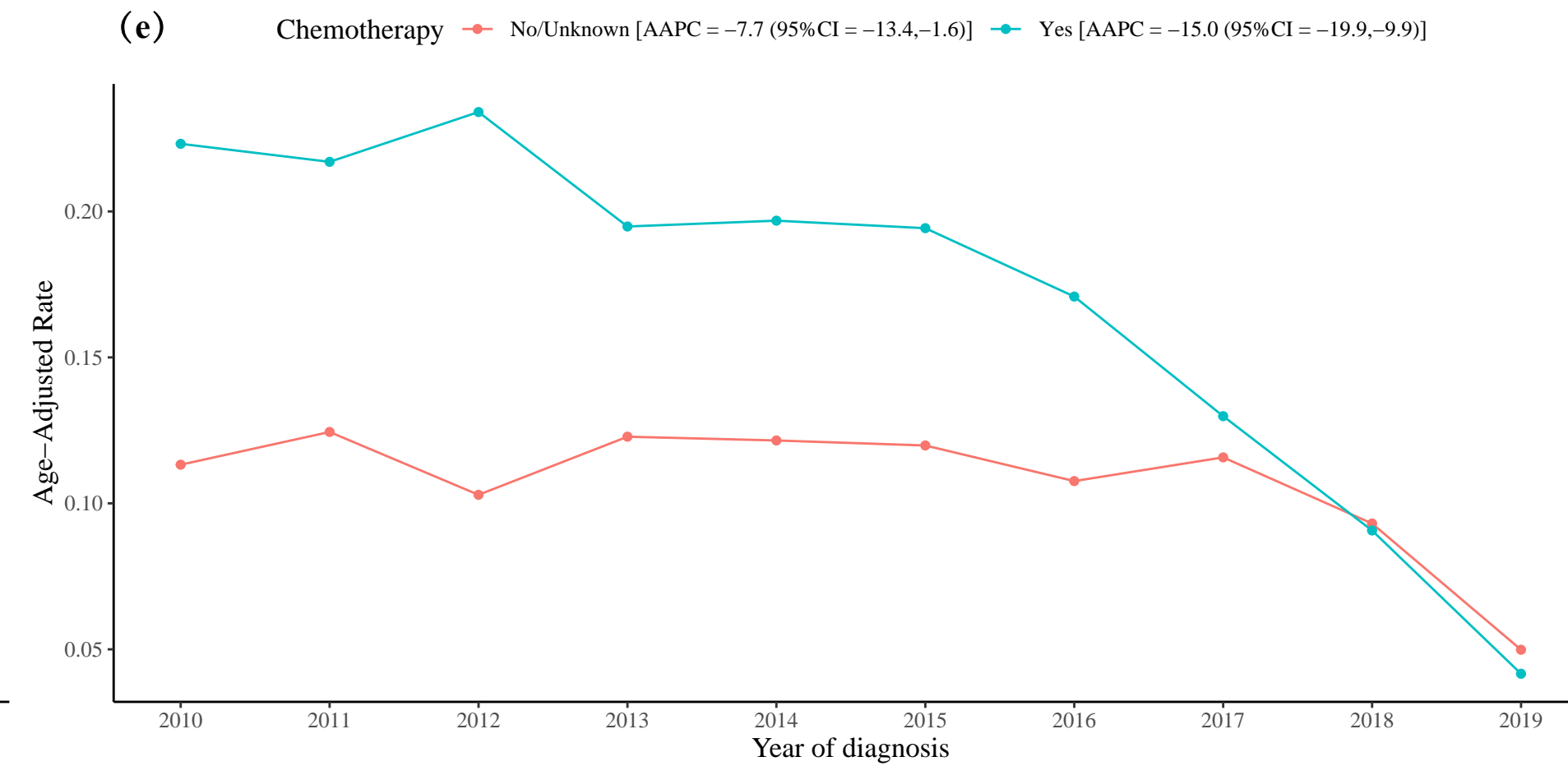

Supplement: S3 Fig — The specific mortality trend of liver metastases in ovarian cancer stratified by (a) age, (b) tumor grade, (c) surgery, (d) radiotherapy and (e) chemotherapy. (PDF) [file pone.0299504.s003.pdf]

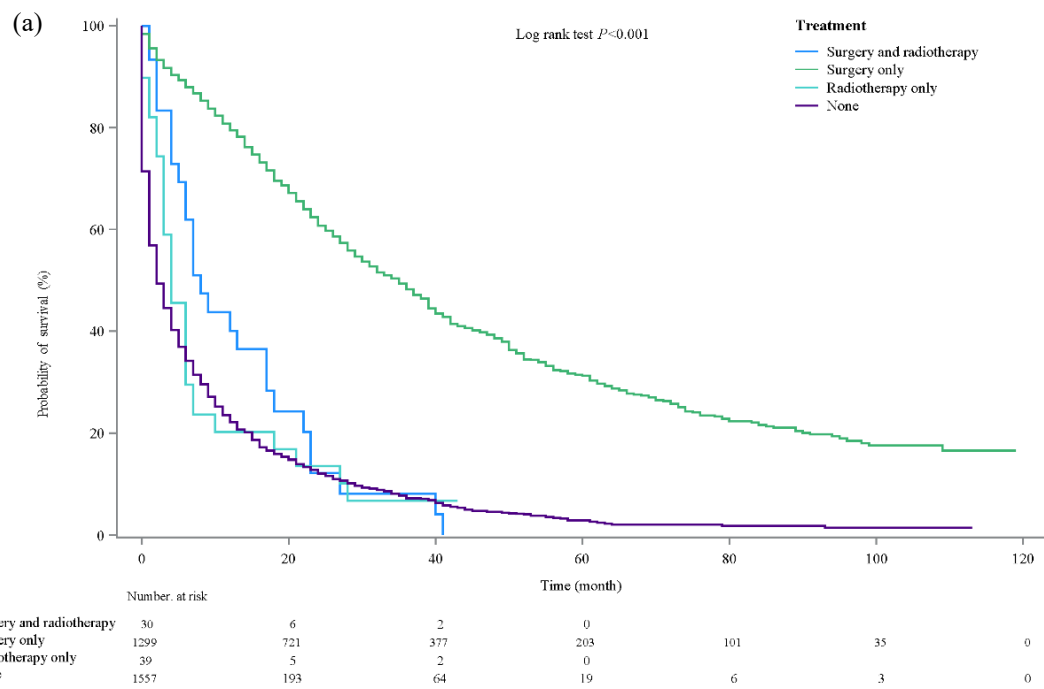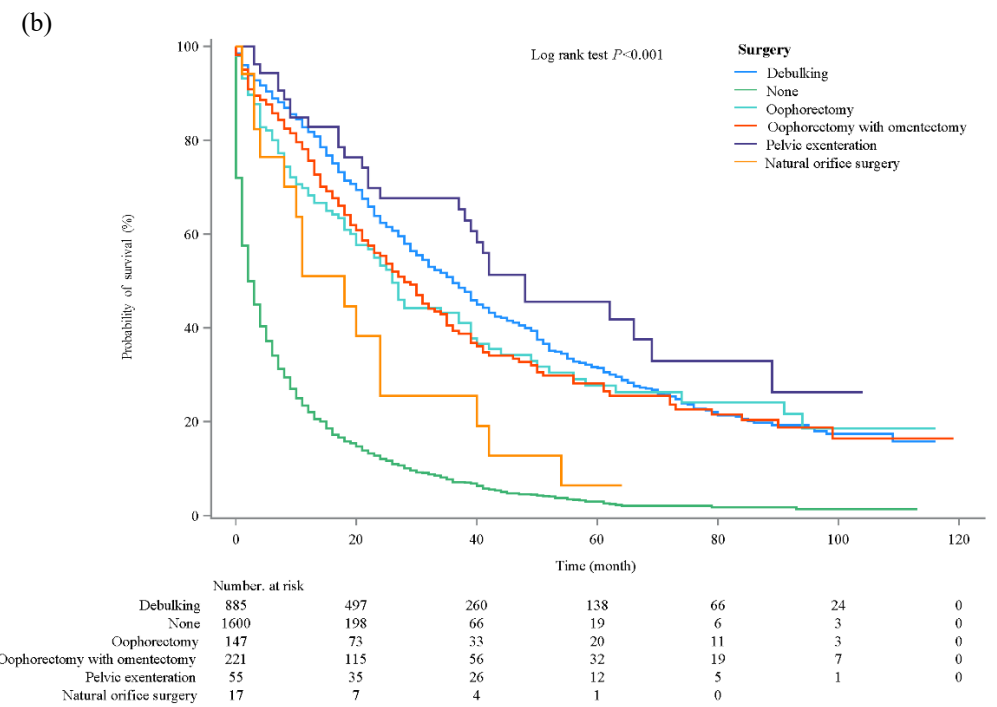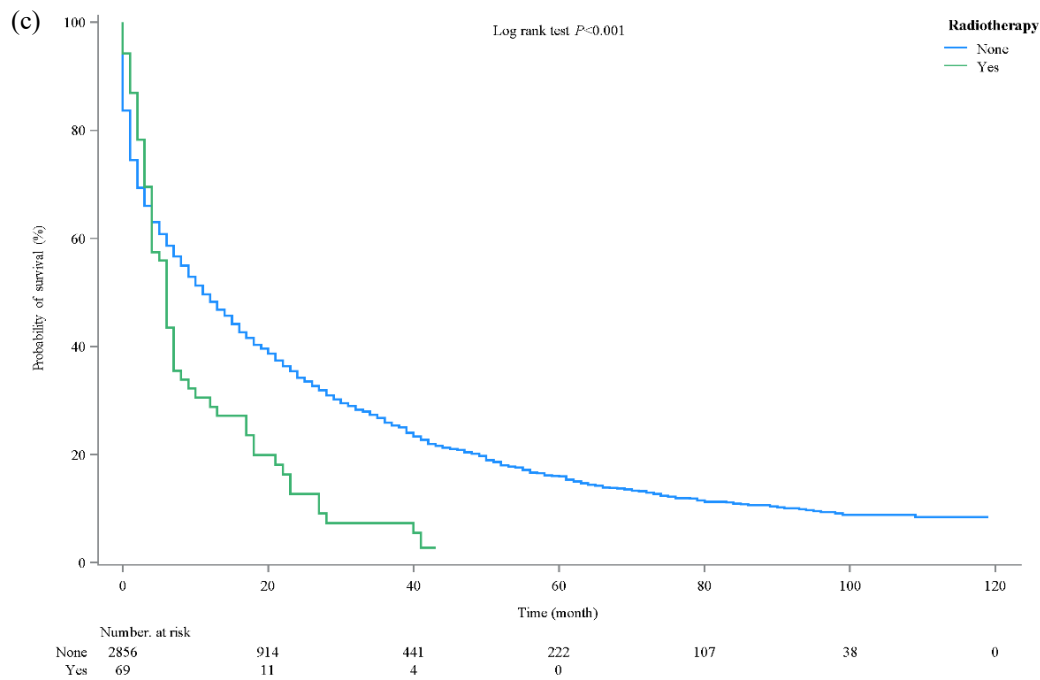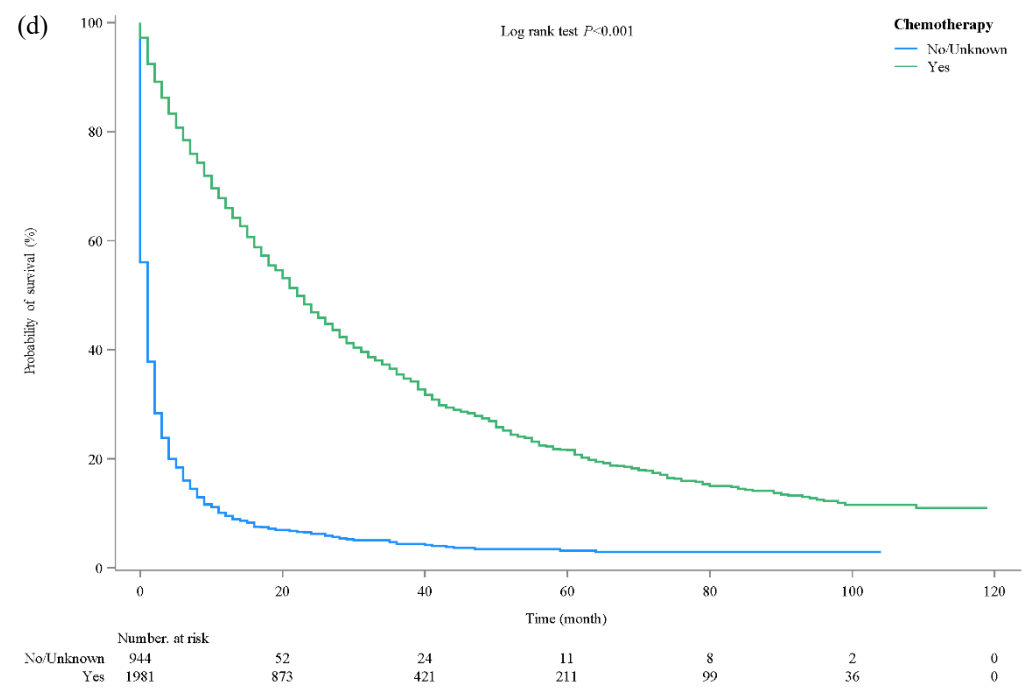

Supplement: S4 Fig — (PDF) [file pone.0299504.s004.pdf]

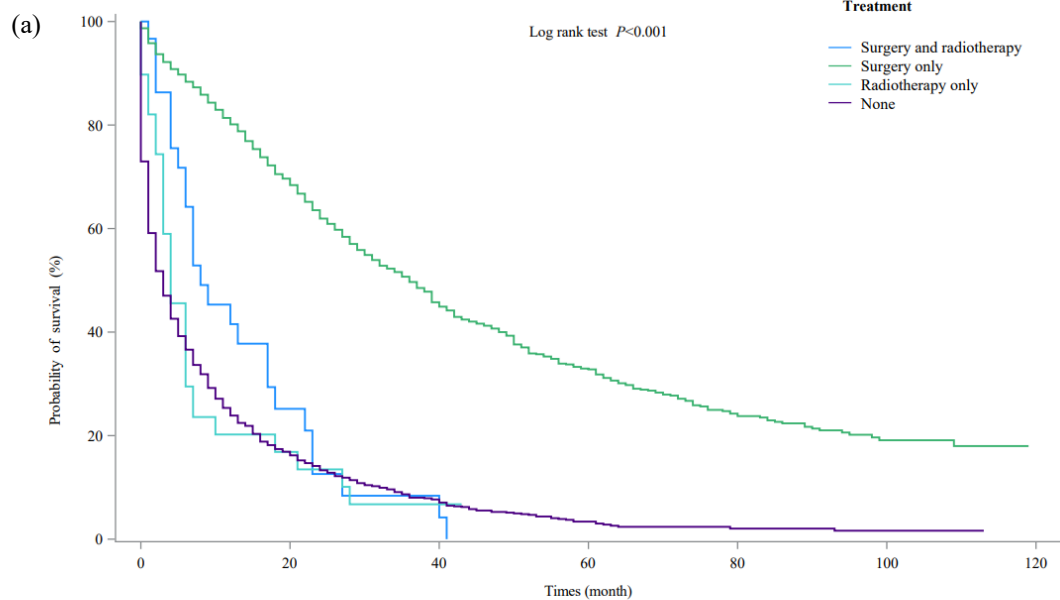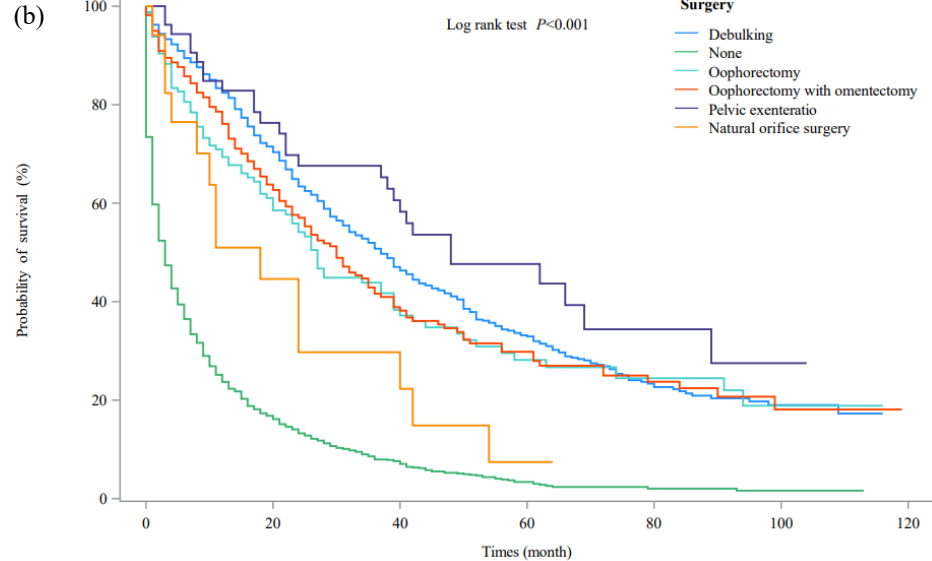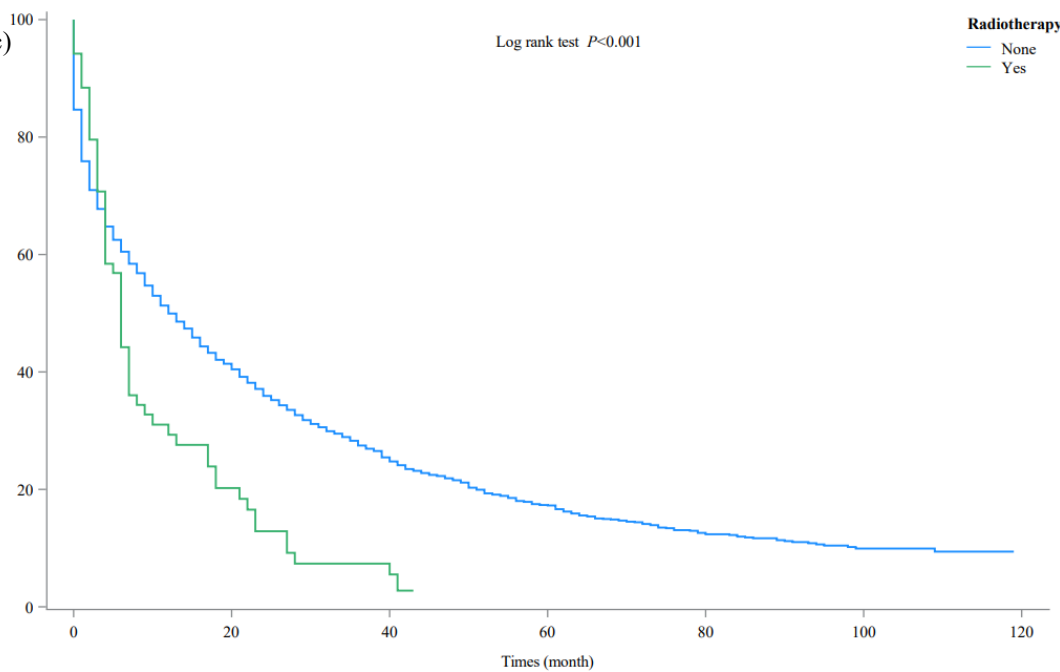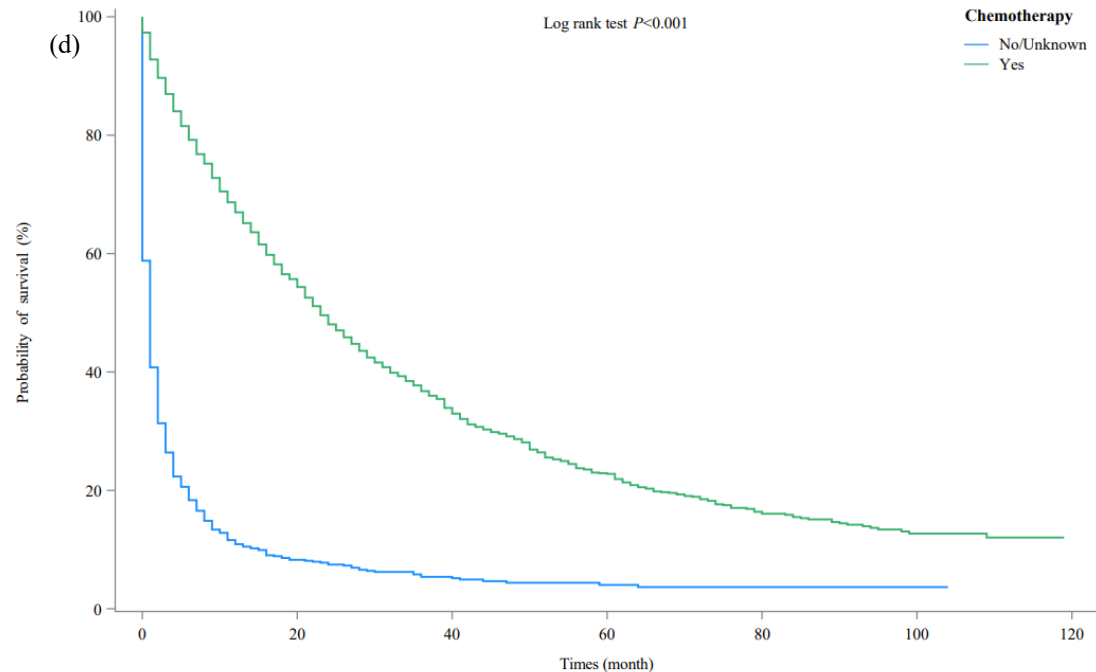

Supplement: S5 Fig — (PDF) [file pone.0299504.s005.pdf]
