# Supplementary material for: Effect of different treatment modalities on ovarian cancer patients with liver metastases: A retrospective cohort study based on SEER
Source: PLoS One. 2024 Apr 18;19(4):e0299504. doi: 10.1371/journal.pone.0299504 (PMC11025763; doi:10.1371/journal.pone.0299504)
Supplement: S1 Table — (DOCX) [file pone.0299504.s006.docx]

**Supplemental Table 1 Screening of confounding factors**

| Variables | OS | | CSS | |
| --- | --- | --- | --- | --- |
|  | HR (95%CI) | *P* | HR (95%CI) | *P* |
| Age | 1.03 (1.02-1.03) | <0.001 | 1.02 (1.02-1.02) | <0.001 |
| Race/ethnicity |  |  |  |  |
| Black | Ref |  | Ref |  |
| White | 0.78 (0.69-0.88) | <0.001 | 0.83 (0.73-0.94) | 0.003 |
| Other | 0.64 (0.53-0.77) | <0.001 | 0.67 (0.56-0.81) | <.001 |
| Marital status |  |  |  |  |
| Married | Ref |  | Ref |  |
| Not married | 1.45 (1.33-1.58) | <0.001 | 1.36 (1.25-1.48) | <0.001 |
| Unknown | 1.21 (0.98-1.50) | 0.080 | 1.11 (0.89-1.38) | 0.377 |
| Income |  |  |  |  |
| <$70,000 | Ref |  | Ref |  |
| ≥ $70,000 | 0.81 (0.74-0.89) | <0.001 | 0.82 (0.75-0.89) | <0.001 |
| Grade |  |  |  |  |
| Grade I & Grade II | Ref |  | Ref |  |
| Grade III& Grade IV | 1.15 (0.90-1.47) | 0.269 | 1.10 (0.86-1.41) | 0.452 |
| Unknown | 2.08 (1.63-2.66) | <0.001 | 1.83 (1.43-2.34) | <0.001 |
| Tumor size |  |  |  |  |
| ≤50 | Ref |  | Ref |  |
| 50-100 | 0.99 (0.85-1.16) | 0.942 | 1.01 (0.87-1.17) | 0.937 |
| 100-200 | 1.11 (0.95-1.29) | 0.196 | 1.09 (0.94-1.27) | 0.244 |
| >200 | 1.52 (1.15-2.00) | 0.003 | 1.54 (1.18-2.03) | 0.002 |
| Unknown | 1.58 (1.38-1.81) | <0.001 | 1.46 (1.29-1.67) | <0.001 |
| Local lymph node metastasis |  |  |  |  |
| No | Ref |  | Ref |  |
| Yes | 1.01 (0.90-1.13) | 0.909 | 1.00 (0.89-1.11) | 0.941 |
| Unknown | 1.26 (1.14-1.39) | <0.001 | 1.13 (1.03-1.25) | 0.013 |
| Histologic |  |  |  |  |
| Carcinosarcoma | Ref |  | Ref |  |
| Clear cell | 0.99 (0.71-1.38) | 0.937 | 1.01 (0.75-1.36) | 0.946 |
| Endometrioid | 0.58 (0.39-0.86) | 0.007 | 0.59 (0.39-0.89) | 0.012 |
| Malignant Brenner Carcinoma | 1.56 (1.25-1.94) | <0.001 | 1.47 (1.19-1.82) | <0.001 |
| Mucinous | 1.83 (1.33-2.53) | <0.001 | 1.83 (1.35-2.48) | <0.001 |
| Serous | 0.55 (0.44-0.69) | <0.001 | 0.57 (0.46-0.70) | <0.001 |
| Other | 1.44 (1.13-1.82) | 0.003 | 1.23 (0.97-1.57) | 0.089 |
| Combined bone metastasis |  |  |  |  |
| No | Ref |  | Ref |  |
| Yes | 1.81 (1.54-2.12) | <0.001 | 1.71 (1.46-2.00) | <0.001 |
| Unknown | 1.56 (1.28-1.90) | <0.001 | 1.47 (1.15-1.87) | 0.002 |
| Combined brain metastasis |  |  |  |  |
| No | Ref |  | Ref |  |
| Yes | 2.30 (1.58-3.34) | <0.001 | 2.49 (1.71-3.64) | <0.001 |
| Unknown | 1.50 (1.24-1.83) | <0.001 | 1.40 (1.10-1.77) | 0.006 |
| Combined lung metastasis |  |  |  |  |
| No | Ref |  | Ref |  |
| Yes | 1.45 (1.32-1.60) | <0.001 | 1.39 (1.27-1.53) | <0.001 |
| Unknown | 1.70 (1.41-2.05) | <0.001 | 1.67 (1.35-2.07) | <0.001 |
| Combined other sites metastasis |  |  |  |  |
| No | Ref |  | Ref |  |
| Yes | 0.95 (0.85-1.06) | 0.369 | 0.92 (0.83-1.03) | 0.166 |
| Unknown | 1.17 (1.01-1.36) | 0.032 | 1.19 (1.02-1.38) | 0.024 |
| CA-125 |  |  |  |  |
| Negative/normal/within normal limits | Ref |  | Ref |  |
| Positive/elevated | 1.06 (0.80-1.39) | 0.695 | 1.15 (0.88-1.50) | 0.318 |
| Unknown | 1.59 (1.19-2.11) | 0.001 | 1.53 (1.16-2.03) | 0.003 |
| Tumor location |  |  |  |  |
| Only one side | Ref |  | Ref |  |
| Bilateral | 1.00 (0.91-1.09) | 0.962 | 1.00 (0.92-1.09) | 0.961 |
| Residual tumor volume |  |  |  |  |
| No gross residual tumor nodules | Ref |  | Ref |  |
| No cytoreductive surgery | 5.83 (4.93-6.90) | <0.001 | 4.66 (3.99-5.44) | <0.001 |
| optimal debulking | 1.52 (1.23-1.88) | <0.001 | 1.51 (1.26-1.81) | <0.001 |
| Residual tumor nodule(s) greater than 1 cm | 1.60 (1.24-2.05) | <0.001 | 1.64 (1.31-2.04) | <0.001 |
| Macroscopic residual tumor nodule(s), size not stated | 2.20 (1.74-2.79) | <0.001 | 2.10 (1.70-2.60) | <0.001 |
| Unknown | 2.11 (1.75-2.55) | <0.001 | 2.01 (1.70-2.39) | <0.001 |

Abbreviations: CA-125=cancer antigen-125; OS=overall survival; CSS=cancer-specific survival;
