# Supplementary material for: Effect of different treatment modalities on ovarian cancer patients with liver metastases: A retrospective cohort study based on SEER
Source: PLoS One. 2024 Apr 18;19(4):e0299504. doi: 10.1371/journal.pone.0299504 (PMC11025763; doi:10.1371/journal.pone.0299504)
Supplement: S2 Fig — The all-cause mortality trend of liver metastases in ovarian cancer stratified by (a) age, (b) tumor grade, (c) surgery, (d) radiotherapy and (e) chemotherapy. (PDF) [file pone.0299504.s002.pdf]

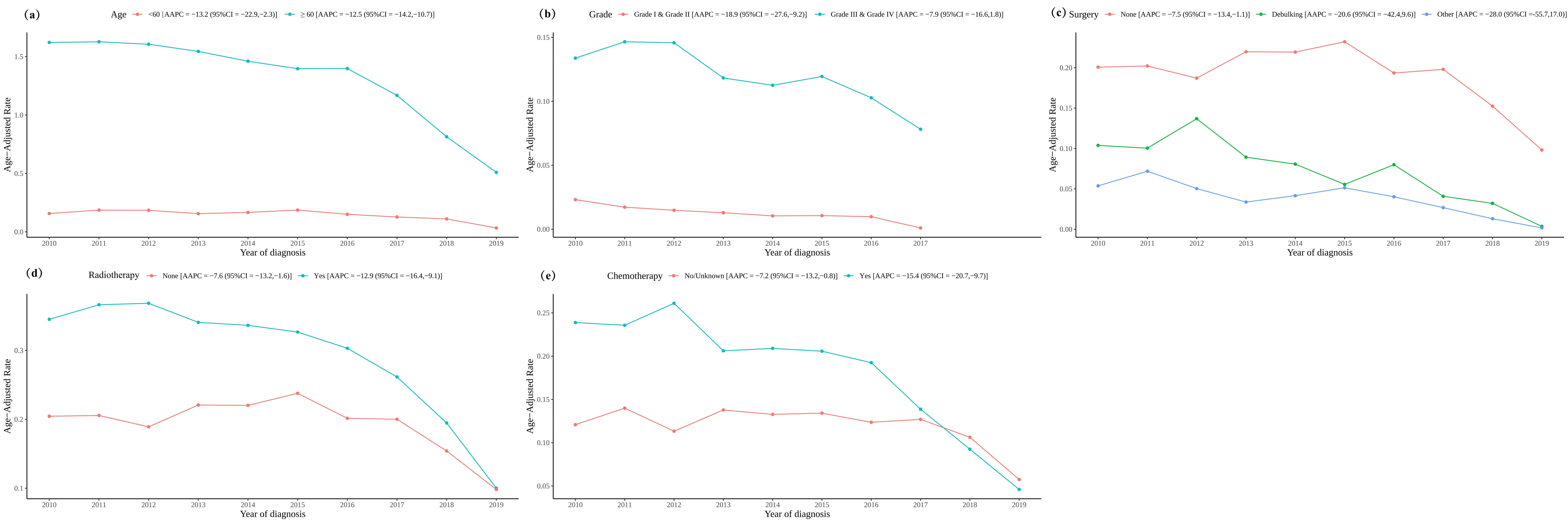
(a)
Age
<60 [AAPC = −13.2 (95%CI = −22.9,−2.3)]
≥ 60 [AAPC = −12.5 (95%CI = −14.2,−10.7)]
Age−Adjusted Rate
Year of diagnosis
(b)
Grade
Grade I & Grade II [AAPC = −18.9 (95%CI = −27.6,−9.2)]
Grade III & Grade IV [AAPC = −7.9 (95%CI = −16.6,1.8)]
Age−Adjusted Rate
Year of diagnosis
(c)
Surgery
None [AAPC = −7.5 (95%CI = −13.4,−1.1)]
Debulking [AAPC = −20.6 (95%CI = −42.4,9.6)]
Other [AAPC = −28.0 (95%CI =−55.7,17.0)]
Age−Adjusted Rate
Year of diagnosis
(d)
Radiotherapy
None [AAPC = −7.6 (95%CI = −13.2,−1.6)]
Yes [AAPC = −12.9 (95%CI = −16.4,−9.1)]
Age−Adjusted Rate
Year of diagnosis
(e)
Chemotherapy
No/Unknown [AAPC = −7.2 (95%CI = −13.2,−0.8)]
Yes [AAPC = −15.4 (95%CI = −20.7,−9.7)]
Age−Adjusted Rate
Year of diagnosis
