# Supplementary material for: Effect of different treatment modalities on ovarian cancer patients with liver metastases: A retrospective cohort study based on SEER
Source: PLoS One. 2024 Apr 18;19(4):e0299504. doi: 10.1371/journal.pone.0299504 (PMC11025763; doi:10.1371/journal.pone.0299504)
Supplement: Attachment — Submitted filename: Response to Reviewers.pdf [file pone.0299504.s008.pdf]

Response Letter

Dear Editor,

Thank you very much for your letter and reviewer's constructive comments on our manuscript entitled "Effect of different treatment modalities on ovarian cancer patients with liver metastases: a retrospective cohort study based on SEER".

In this revised version, changes to our manuscript were highlighted by red colored text, and point by point responses to reviewer are listed below this letter.

Looking forward to your reply.

Yours sincerely,

Bona Liu

Additional Editor Comments:

Dear Authors,

I read with interest your study.

As a further comment I would like to understand the treatment modalities you have included in your analysis. Do you have considered only Primary debulking surgery and excluded Neoadjuvant chemotherapy followed by interval debulking surgery? If this is the case you should state about it in the discussion, in fact resection of liver metastasis is feasible also after chemotherapy. Otherwise, if you included these patients you have to definetly reorganize the manuscript highlighting the treatment modalities. In both the option, I suggest to refer to https://www.ejgo.net/articles/10.31083/j.ejgo4301015, so you can either state this limit or clarify the treatment modalities.

Many thanks.

Federico Ferrari, MD, PhD

Answer: Thanks very much for your advice. We agree with you. Since all the data in this retrospective cohort study were derived from the SEER database, we could not distinguish the sequence of chemotherapy and surgery in patients; that is, we could not distinguish between PDS and IDS. This is also one of the limitations described in the discussion section. Further prospective studies should be conducted to confirm our findings. At the same time, we also describe in the discussion section the fact that resection of liver metastases after chemotherapy is also feasible [1]. Please see the revised manuscript.

[1] Forte S, Ferrari F, Valenti G, Capozzi VA, Santana BN, Babin G, Guyon F. Liver surgery for advanced ovarian cancer: a systematic review of literature. Eur. J. Gynaecol. Oncol. 2022; 43(1): 64–72. doi: http://doi.org/10.31083/j.ejgo4301015
